# Vitamin D and exercise in obesity: a neurovascular–muscle axis

**DOI:** 10.3389/fnut.2026.1750915

**Published:** 2026-03-12

**Authors:** Xiaoxia Zheng, Chuanlong Zhang

**Affiliations:** Department of Physical Education, Luoyang Institute of Science and Technology, Luoyang, Henan, China

**Keywords:** adipose tissue inflammation, exercise modality, insulin resistance, metabolic inflexibility, neurocognitive health, neurovascular–muscle axis, obesity-related complications, skeletal muscle adaptation

## Abstract

Obesity is characterized by chronic low-grade inflammation, insulin resistance, impaired skeletal muscle function, and disturbances in neurovascular health. Metabolic inflexibility, defined as a reduced capacity to appropriately switch between lipid and glucose utilization in response to physiological demands, represents a central pathophysiological feature linking these alterations. Emerging evidence suggests that physical exercise and vitamin D status influence overlapping molecular pathways involved in energy metabolism, inflammation, vascular function, and neural signaling. Exercise robustly improves mitochondrial function, endothelial health, and myokine-mediated cross-talk between muscle, adipose tissue, and the brain, while vitamin D, acting through the vitamin D receptor (VDR), modulates calcium homeostasis, immune signaling, and tissue-specific metabolic responsiveness. This narrative review synthesizes mechanistic and translational evidence on how vitamin D and exercise may interact within a neurovascular–muscle axis to influence metabolic regulation, adipose inflammation, skeletal muscle adaptation, and neurocognitive function in obesity. Importantly, current evidence supports exercise as the primary driver of metabolic improvement, whereas vitamin D may exert context-dependent, adjunctive effects, particularly in deficient populations. Rather than proposing a standalone therapy, this review situates the vitamin D–exercise interaction as a complementary strategy that may enhance functional and systemic adaptations relevant to obesity-related complications. Limitations related to causal inference and population heterogeneity are highlighted, underscoring the need for well-powered, obesity-specific clinical trials to clarify translational relevance.

## 1 Introduction

Obesity represents a multifaceted condition of considerable complexity, characterized not solely by an excess of adipose tissue but also by significant disruptions in metabolic, inflammatory, musculoskeletal, and neurocognitive domains of health. A primary characteristic of obesity is the presence of chronic low-grade systemic inflammation, predominantly instigated by hypertrophied adipocytes and aberrant immune cell infiltration, particularly involving M1-polarized macrophages, which collectively foster increased production of cytokines (1, 2). This inflammatory environment directly exacerbates insulin resistance, hindering glucose uptake in skeletal muscle, adipose tissue, and hepatic cells via the perturbation of insulin receptor signaling cascades (3, 4). Obesity can coexist with sarcopenic obesity, a pathological state defined by the concurrent presence of excessive adiposity alongside the degradation of muscle mass, quality, and functional strength, which further intensifies metabolic dysfunction and diminishes physical performance and overall quality of life (5, 6). Furthermore, the metabolic and vascular derangements associated with obesity have repercussions that extend to the central nervous system, might contribute to neurocognitive decline, compromised neurovascular integrity, and an augmented susceptibility to dementia (7, 8). It should be noted that the association between low circulating vitamin D levels and obesity is largely correlational, and may reflect volumetric dilution, adipose sequestration, or behavioral confounding rather than a direct causal role of vitamin D deficiency in obesity pathogenesis.

Obesity is increasingly recognized as a disorder involving central metabolic dysregulation in addition to peripheral adipose and skeletal muscle dysfunction (9). Neuroimaging studies in humans demonstrate structural and functional alterations in the hypothalamus, including gliosis and inflammatory signaling, which may impair leptin and insulin responsiveness and disrupt central energy homeostasis. Beyond hypothalamic changes, individuals with obesity exhibit altered cerebral glucose metabolism in cortical and subcortical regions involved in appetite regulation, rewards processing, and executive function (9). Evidence of central insulin resistance further suggests that metabolic impairment extends to neural tissue, influencing not only appetite control but also neurovascular coupling, mitochondrial efficiency, and synaptic plasticity (10). Chronic low-grade systemic inflammation, a hallmark of obesity, may exacerbate these alterations through microglial activation and endothelial dysfunction, potentially compromising cerebral perfusion and metabolic flexibility (10). These central disturbances provide an important neurobiological context for understanding how vitamin D signaling and exercise, both of which influence inflammatory tone, endothelial function, and insulin sensitivity, may interact within an integrated neurovascular–muscle framework (9).

**FIGURE 1:**
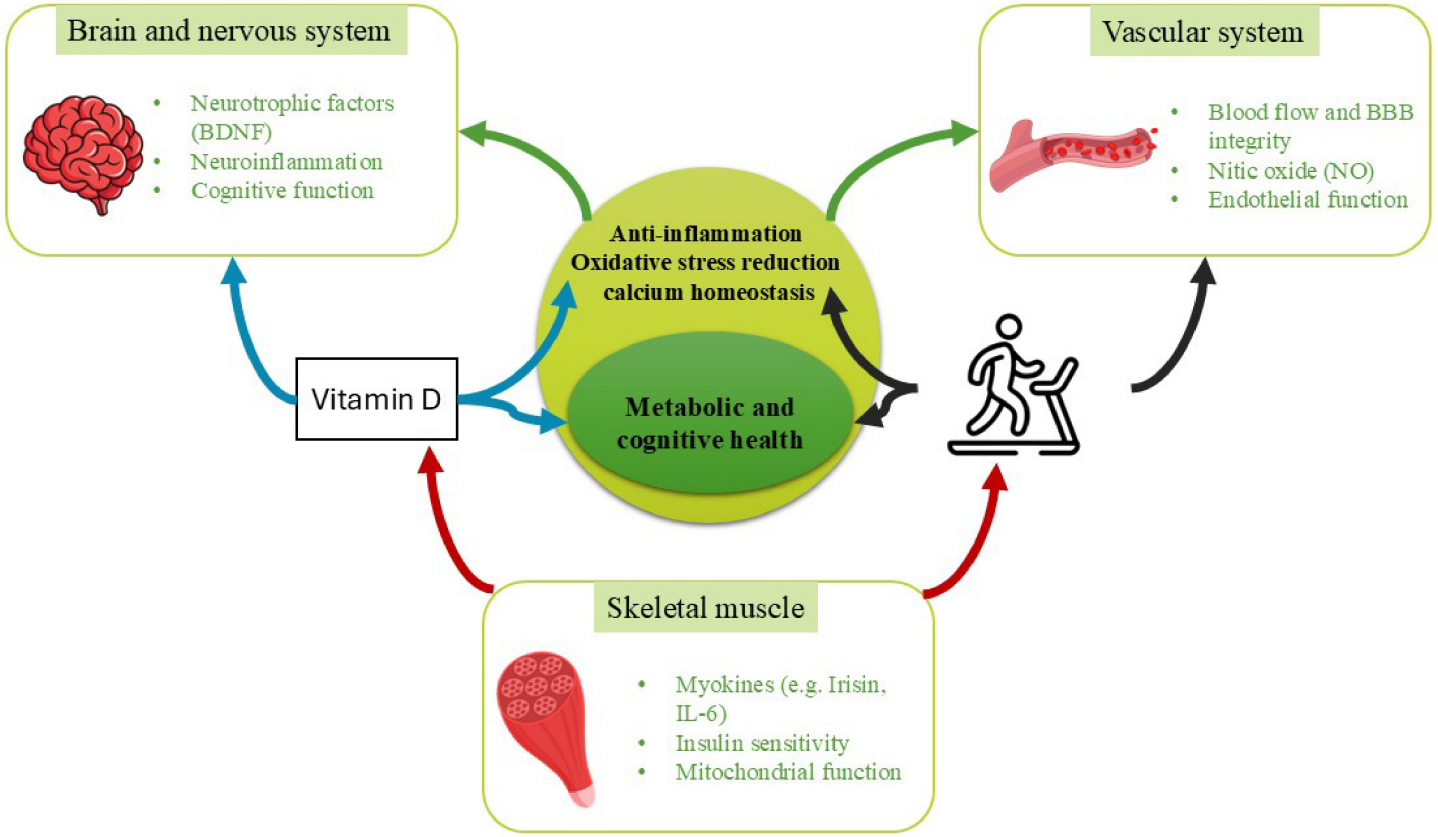
Conceptual model of the neurovascular–muscle axis in obesity and its modulation by vitamin D and exercise. This schematic illustrates the proposed neurovascular–muscle axis as an integrative framework linking skeletal muscle metabolism, vascular function, and neurocognitive health in the context of obesity. Skeletal muscle communicates with the vascular and nervous systems through exercise-induced myokines (e.g., IL-6, irisin), improvements in mitochondrial function, and enhanced insulin sensitivity. The vascular system modulates tissue perfusion, endothelial nitric oxide (NO) signaling, and blood–brain barrier (BBB) integrity, thereby influencing both muscle metabolism and neural function. Neural components include neurotrophic signaling (e.g., BDNF), neuroinflammation, and cognitive performance, which are sensitive to metabolic and vascular inputs. Vitamin D and physical exercise act as convergent modulators across all three domains, influencing inflammation, oxidative stress, calcium homeostasis, and mitochondrial biogenesis via VDR-dependent and activity-dependent pathways. Solid arrows denote interactions supported by empirical evidence in obesity-related animal and human studies, whereas dashed arrows represent inferred or emerging bidirectional links that remain to be directly validated in integrated experimental models. The axis is presented as a hypothesis-driven construct intended to synthesize existing evidence and guide future mechanistic and clinical research.

In order to gain a more profound comprehension of the systemic characteristics of these abnormalities, we use a neurovascular–muscle axis framework to integrate a theoretical construct that elucidates the bidirectional interactions between neural functionality, vascular integrity, and skeletal muscle metabolism. This axis (Figure 1) encompasses neurotrophic signaling, cerebrovascular perfusion, endothelial functionality, mitochondrial integrity, and muscle-derived myokines, all of which play a crucial role in maintaining metabolic homeostasis and supporting brain health (11–13). Disruptions in any singular component, whether neural, vascular, or muscular, may induce dysfunction throughout the entire system, particularly in individuals with overweight or obesity, where inflammation and metabolic disturbances are notably prevalent (14).

Within this paradigm, vitamin D and physical exercise are increasingly acknowledged as pivotal modulators that can concurrently impact neurovascular and muscular pathways (15–17). Vitamin D fulfills critical functions in insulin sensitivity, skeletal muscle performance, immunomodulation, and the maintenance of endothelial integrity via its receptor (VDR), which is extensively expressed in metabolic and neurovascular tissues (17–19). Conversely, exercise serves as one of the most potent physiological stimuli for augmenting skeletal muscle metabolism, mitigating adipose inflammation, enhancing vascular function, and fostering neuroplasticity through mechanisms that encompass myokines, improved mitochondrial dynamics, and increased cerebral perfusion (20, 21). Recent empirical findings indicate that vitamin D status may influence the adaptations induced by exercise, while exercise may, in turn, enhance vitamin D metabolism and tissue responsiveness, thereby suggesting the existence of a synergistic interaction that could amplify therapeutic outcomes (22, 23).

This review endeavors to meticulously encapsulate the extant literature regarding the synergistic influences of vitamin D and physical exercise within the framework of the neurovascular–muscle axis, with a concentrated emphasis on four pivotal domains that are particularly pertinent to individuals with overweight and obesity: adipose tissue inflammation, insulin resistance alongside metabolic regulation, skeletal muscle metabolism coupled with sarcopenic obesity, and the interrelations between neurovascular integrity and cognitive health. By amalgamating mechanistic elucidations with empirical clinical insights, this review underscores the potential of vitamin D–exercise interplay as an innovative therapeutic approach aimed at mitigating obesity-related pathologies and enhancing multifaceted health outcomes. This article is intended as a narrative, hypothesis-driven review rather than a systematic review or meta-analysis. The literature was identified through targeted searches of PubMed, Web of Science, and Scopus using combinations of keywords related to vitamin D, exercise, obesity, insulin resistance, skeletal muscle, inflammation, and neurovascular or cognitive outcomes. Studies were selected to illustrate mechanistic pathways, translational links, and representative clinical findings across animal and human models. No formal inclusion or exclusion criteria or quantitative quality assessment was applied. Consequently, the presented evidence should be interpreted as a conceptual synthesis rather than an exhaustive or statistically weighted evaluation of all available studies.

## 2 Molecular and physiological basis of the vitamin D–exercise interaction

Most evidence for this interaction derives from cell culture and animal models, with limited and heterogeneous confirmation in populations with obesity. To maintain conceptual consistency with Figure 1, interactions described throughout the following sections are classified according to the strength and population specificity of available evidence. Relationships supported by direct human evidence in populations with overweight or obesity are described as empirically supported. Interactions demonstrated in human studies but not specifically within obesity populations are described as supported in related human contexts. Mechanistic relationships derived primarily from animal models, cellular studies, or indirect physiological inference are identified as mechanistically inferred. This framework is applied systematically across molecular, metabolic, and functional domains to clarify which components of the proposed neurovascular–muscle axis is established versus hypothesized within obesity-specific pathophysiology.

### 2.1 Vitamin D metabolism and VDR signaling in muscle, brain, and adipose tissue

Vitamin D undergoes a series of hydroxylation processes in the liver and kidneys, resulting in the synthesis of its biologically active form, 1,25-dihydroxyvitamin D3 [1,25(OH)2D3], which mediates its physiological functions via the vitamin D receptor (VDR), a nuclear transcription factor that is expressed in skeletal muscle, the brain, adipose tissue, and vascular endothelium (Figure 1) (18, 24). In skeletal muscle, VDR is instrumental in the regulation of calcium homeostasis, mitochondrial biogenesis, synthesis of contractile proteins, and the differentiation of myogenic cells (25). A deficiency in vitamin D is correlated with diminished muscle strength, decreased oxidative capacity, and an increase in intramuscular fat accumulation (26, 27). In adipose tissue, the activation of VDR exerts an influence on processes such as adipogenesis, lipolysis, the secretion of adipokines, and the polarization of macrophages, frequently resulting in the suppression of pro-inflammatory gene expression while simultaneously fostering metabolic equilibrium (28). In the brain, VDR signaling plays a critical role in the modulation of neurotrophic pathways, the maintenance of neurovascular integrity, glucose transport, and the regulation of immune responses, with deficiencies being associated with cognitive decline, neuroinflammation, and compromised neurovascular coupling (29). Although low vitamin D status has been consistently associated with cognitive impairment and neuroinflammation, current human evidence does not definitively establish causality, and observed relationships may be influenced by age, comorbidity burden, physical activity, and overall metabolic health.

### 2.2 Exercise-induced regulation of vitamin D activation and receptor expression

Exercise interacts with the metabolism of vitamin D at various physiological levels. Both aerobic and resistance training modalities have been demonstrated to elevate circulating levels of 25(OH)D, partially due to enhanced mobilization from adipose tissue reserves, improved hepatic metabolic processes (30, 31). Furthermore, exercise promotes the upregulation of vitamin D receptor (VDR) expression in skeletal muscle, thereby augmenting the tissue’s sensitivity to vitamin D and fostering enhanced mitochondrial and anabolic adaptations (32, 33). In addition, physical activity may enhance vascular perfusion and endothelial functionality, thereby improving the transport of vitamin D metabolites to target tissues while concurrently mitigating the obesity-related sequestration of vitamin D within adipose tissue (33, 34). These mechanisms indicate that physical activity may enhance the biological availability and signaling efficacy of vitamin D across various organ systems.

### 2.3 Converging molecular pathways: AMPK, PGC-1α, NF-κB, PI3K/Akt, and myokines

The synergistic interactions between vitamin D and physical activity may emerge from their engagement with various pivotal metabolic and inflammatory pathways. Exercise-induced activation of AMPK and PGC-1α is empirically supported in humans with overweight and obesity, where it contributes to improved mitochondrial biogenesis and insulin sensitivity. In contrast, vitamin D–mediated modulation of these pathways during exercise is primarily mechanistically inferred from animal models and deficiency-focused human studies (35). Vitamin D attenuates inflammation mediated by NF-κB, leading to a decrease in cytokine production within adipose and muscular tissues, whereas physical activity independently diminishes NF-κB activity and elevates the levels of anti-inflammatory myokines such as IL-6, IL-10, and irisin (36, 37). Exercise-induced enhancement of PI3K/Akt signaling and glucose uptake is well supported in human obesity cohorts. Vitamin D–related modulation of PI3K/Akt signaling, particularly in the context of exercise training, remains supported in related human contexts but is largely mechanistically inferred in obesity-specific populations (38, 39). Myokines released as a consequence of exercise particularly irisin, BDNF, myostatin inhibitors, and IL-6 engage with VDR-regulated pathways to improve metabolic flexibility, mitigate oxidative stress, and alter neuroplasticity (40). Taken together, these mechanisms elucidate that the status of vitamin D can significantly influence the extent of adaptations induced by exercise and, conversely, the effects of exercise can modify vitamin D status. Notably, aerobic exercise preferentially activates AMPK–PGC-1α–dependent mitochondrial and fatty acid oxidation pathways, resistance training primarily engages mTOR signaling and myostatin suppression to promote muscle anabolism, and HIIT may enhance metabolic flexibility and insulin sensitivity, with vitamin D–VDR signaling potentially modulating the magnitude of these modality-specific adaptations.

### 2.4 The neurovascular–muscle axis as a unifying mechanism

The neurovascular–muscle axis presents a coherent conceptual framework for understanding the interaction between vitamin D and exercise. This axis establishes a connection among neuronal activity, vascular perfusion, and the metabolic processes within skeletal muscle via endocrine, paracrine, and neural feedback mechanisms (41, 42). The neurovascular–muscle axis refers to the interconnected relationship between the nervous system, vascular health, and skeletal muscle metabolism, where disruptions in one component can influence the others, particularly in conditions such as obesity and metabolic disease. This axis involves neurovascular integrity, where brain signaling pathways influence vascular function and, in turn, impact muscle metabolism (43). Exercise and physical activity play a crucial role in modulating this axis by enhancing blood flow, improving endothelial function, and promoting the release of myokines, which are signaling molecules produced by muscle during contraction. These myokines have direct effects on the brain and vasculature, fostering neuroprotection, reducing inflammation, and supporting metabolic homeostasis. Disruptions in this axis, especially through chronic inflammation or impaired vascular function, contribute to metabolic dysfunction, muscle atrophy, and cognitive decline commonly observed in obesity-related disorders. As a result, understanding this axis may lead to novel therapeutic strategies that target multiple systems to improve health outcomes, particularly in individuals with overweight or obesity (41, 43, 44).

**FIGURE 2:**
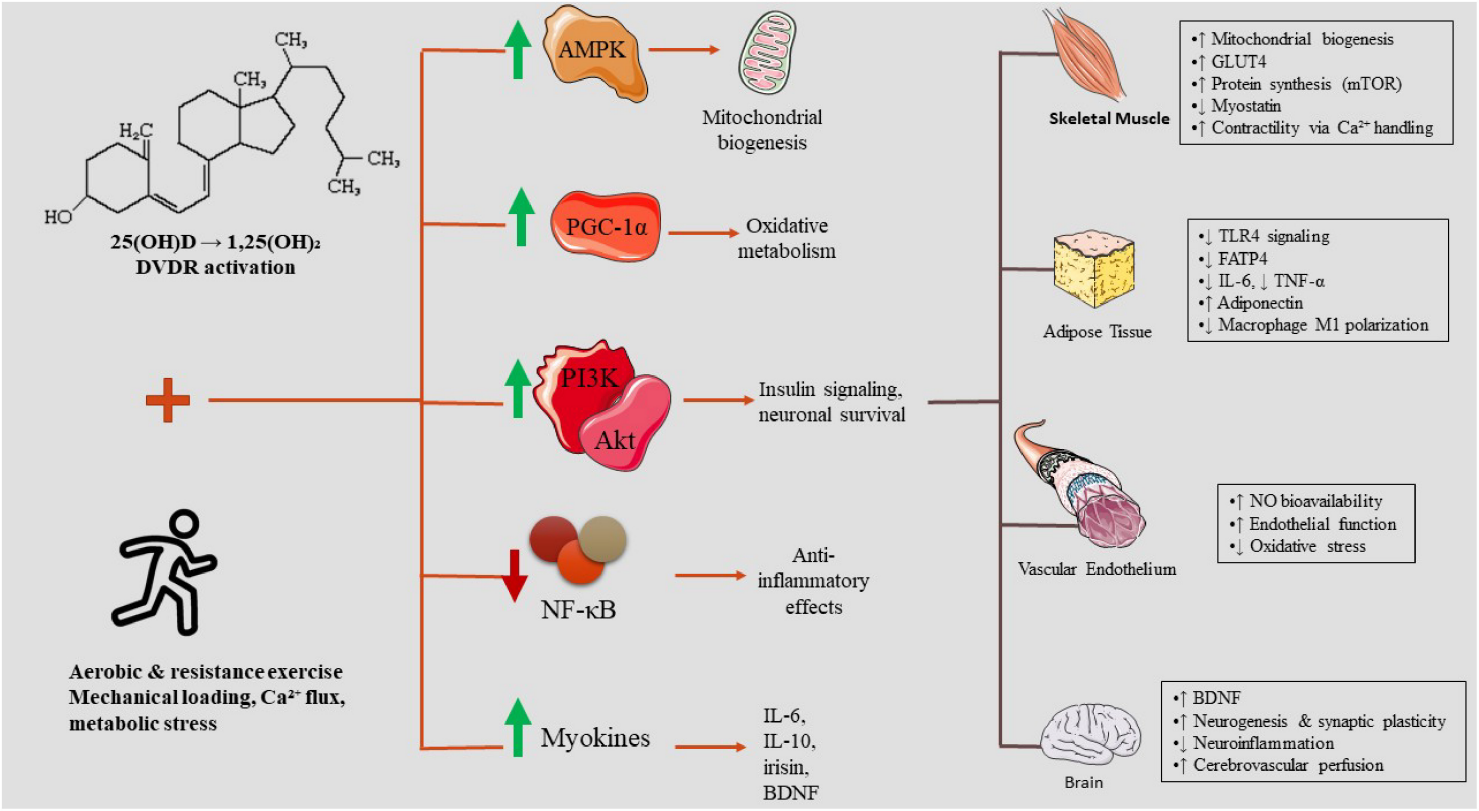
Integrated mechanisms through which vitamin D and exercise converge on shared molecular pathways to influence muscle, adipose tissue, vasculature, and brain. Vitamin D (via VDR activation) and exercise (via calcium flux, mechanical load, metabolic stress, and myokines) jointly activate AMPK, PGC-1α, PI3K/Akt, and anti-inflammatory pathways while suppressing NF-κB. These combined signals enhance muscle mitochondrial biogenesis, GLUT4 expression, and protein synthesis; reduce adipose inflammation through lowered TLR4, FATP4, TNF-α, and IL-6; improve endothelial NO signaling and vascular function; and increase brain BDNF, neurogenesis, and neurovascular integrity. The coordinated effects across tissues illustrate the neurovascular–muscle axis linking metabolic, inflammatory, and cognitive outcomes.

Vitamin D plays a pivotal role in maintaining neurovascular integrity by modulating the production of endothelial nitric oxide, ensuring the stability of the blood–brain barrier, and influencing the expression of neurotrophins (45). The impact of exercise amplifies these effects through the enhancement of cerebrovascular circulation, the stimulation of angiogenesis, and the elevation of neurotrophic factors such as brain-derived neurotrophic factor (BDNF) (46, 47). Within skeletal muscle, the combined effects of vitamin D and exercise may facilitate mitochondrial efficiency and mitigate local inflammation, which subsequently diminishes systemic inflammatory burden and enhances adipose tissue functionality. These declines in inflammation and advancements in metabolic health further bolster neurocognitive performance, thereby establishing a reciprocal relationship throughout the neurovascular–muscle axis (41, 48). Consequently, vitamin D and exercise collaboratively optimize energy metabolism, vascular equilibrium, and neural functionality, thereby constituting an integrated therapeutic approach for individuals with overweight or obesity (Figure 2).

To maintain conceptual consistency with Figure 1, interactions described throughout the following sections are explicitly classified according to the strength and population specificity of available evidence. Relationships supported by direct human evidence in populations with overweight or obesity are described as empirically supported (corresponding to solid lines in Figure 1). Interactions demonstrated in human studies but not specifically within obesity populations are described as supported in related human contexts. Mechanistic relationships derived primarily from animal models, cellular studies, or indirect physiological inference are identified as mechanistically inferred (corresponding to dashed lines in Figure 1). This framework is applied systematically across molecular, metabolic, and functional domains to clarify which components of the proposed axis are established versus hypothesized within obesity-specific pathophysiology.

However, whether these mechanistic overlaps translate into additive or synergistic clinical benefit in humans with obesity remains unproven, as deficiency-stratified, adequately powered trials with standardized exercise dose and clinically meaningful endpoints are lacking. Within obesity-specific contexts, several components of this axis are supported by human evidence, including exercise-induced improvements in insulin sensitivity, endothelial function, adipose inflammation, and skeletal muscle metabolic capacity. By contrast, direct evidence demonstrating coordinated vitamin D–exercise interactions across neural, vascular, and muscular systems in individuals with obesity remains limited. The proposed integrative signaling relationships involving neurotrophic regulation, cerebrovascular coupling, and vitamin D–mediated modulation of exercise responsiveness are therefore primarily mechanistically inferred from preclinical models or extrapolated from non-obesity human populations. Accordingly, the neurovascular–muscle axis should be interpreted as a conceptual framework that organizes converging biological pathways rather than as a fully empirically validated systems model in obesity.

Recent integrative evidence from expands on canonical molecular pathways by illustrating how vitamin D and physical exercise converge on shared neurotrophic and anti-inflammatory signaling networks relevant to neuroplasticity and muscle metabolism. Chen et al. (15) provide a comprehensive synthesis of preclinical and clinical data demonstrating that combined vitamin D and exercise interventions up-regulate neurotrophic factors such as BDNF and IGF-1 and enhance vascular endothelial growth factor (VEGF) expression, which we now situate alongside established mechanisms of AMPK–SIRT1/PGC-1α and NF-κB modulation (Figure 1). This convergence suggests that the modulation of oxidative stress and cytokine profiles by vitamin D and exercise is not simply additive but targets overlapping intracellular effectors that support neurovascular coupling and metabolic resilience. In parallel, Farina et al. (49) highlights that vitamin D’s genomic and non-genomic actions, via VDR and rapid signaling pathways, attenuate pro-inflammatory cascades implicated in neurodegeneration, reinforcing the role of systemic anti-inflammatory tone in the proposed neurovascular–muscle axis. Together, these studies provide mechanistic depth to the argument that vitamin D status can shape both central nervous system plasticity and peripheral metabolic flexibility in response to physical activity. Importantly, Chen et al. (15) emphasize that synergistic neuroprotective effects are most consistently observed in preclinical models and aging cohorts, whereas human interventional trials remain heterogeneous and underpowered. This observation parallels the translational uncertainty noted in obesity-specific metabolic trials throughout the present review. Thus, while molecular convergence is biologically plausible, clinical synergy remains conditional and population-dependent. Incorporating these findings into the neurovascular–muscle axis model underscores that central neuroplastic adaptations may be more sensitive to combined interventions than peripheral metabolic endpoints, suggesting differential tissue responsiveness across the axis. Complementarily, Farina and Crescioli (49) foreground redox-sensitive transcriptional regulators such as Nrf2, proposing that vitamin D sufficiency may prime antioxidant gene expression and thereby enhance exercise-induced mitochondrial resilience. Compared to earlier obesity-centered inflammatory models, these perspectives broaden the axis to include oxidative stress buffering and synaptic preservation, reinforcing, but also complicating, the proposed integrative framework.

## 3 Modulation of adipose inflammation and insulin resistance

Evidence for overlap in these signaling pathways is strongest in cellular and animal models, with human data largely derived from exercise interventions and limited direct evaluation of vitamin D–specific modulation in populations with obesity. While exercise robustly activates these pathways and improves metabolic and inflammatory profiles, vitamin D–related effects are more variable and appear dependent on baseline deficiency, tissue context, and outcome measured. Importantly, acute exercise-induced cytokine responses (e.g., IL-6 acting as a myokine) should not be conflated with chronic low-grade inflammation characteristic of obesity. At present, it remains uncertain whether these shared molecular pathways translate into clinically meaningful additive or synergistic effects in humans with obesity, as well-powered, deficiency-stratified randomized trials with standardized vitamin D dosing, defined exercise prescriptions, and relevant metabolic or functional endpoints are still lacking. Human exercise trials support activation of this pathway, whereas vitamin D–specific modulation remains largely inferential. Consistent with the evidence-classification framework introduced above, improvements in insulin signaling, adiposity, and inflammatory markers are strongly supported by human exercise interventions in populations with obesity. Evidence for vitamin D–specific modulation of these pathways in obesity is comparatively limited and heterogeneous, with most mechanistic support derived from animal studies or human populations selected for deficiency rather than adiposity *per se*. Proposed synergistic effects involving coordinated regulation of AMPK, NF-κB, and PI3K/Akt signaling therefore remain mechanistically plausible but not definitively established in obesity-specific clinical settings. Improvements in insulin signaling, adipose inflammatory tone, and endothelial function in response to exercise are empirically supported in human obesity cohorts and therefore represented as validated (solid-line) interactions in Figure 1.

To maintain consistency with the evidence-classification framework introduced in Section “2 Molecular and physiological basis of the vitamin D–exercise interaction,” mechanistic interactions discussed in this section are explicitly identified as (i) empirically supported in humans with obesity, (ii) supported in related human contexts, or (iii) mechanistically inferred from animal, cellular, or indirect physiological evidence.

### 3.1 Combined effects of vitamin D and aerobic/resistance exercise on adipose inflammation

Obesity is defined by a state of persistent low-grade inflammation that is primarily facilitated by the presence of hypertrophic adipocytes, the increased infiltration of pro-inflammatory M1 macrophages, and a dysregulated secretion of adipokines. This inflammatory milieu fosters elevated concentrations of TNF-α, IL-6, and various other cytokines that disrupt metabolic homeostasis (2). Both vitamin D and physical exercise have been shown to independently mitigate these inflammatory responses; however, an expanding the combined synergistic anti-inflammatory effect still remains a hypothesis. Vitamin D exerts anti-inflammatory effects through inhibition of NF-κB signaling and modulation of macrophage polarization; however, in obesity-specific human cohorts, these effects are primarily mechanistically inferred from preclinical models and cellular studies (50, 51). Concurrently, vitamin D may enhance the synthesis of IL-10, a cytokine that plays a pivotal role in limiting adipose tissue inflammation and reinstating tissue homeostasis (52). Both aerobic and resistance training modalities have been shown to diminish adipose inflammatory markers through the reduction of visceral fat mass, enhancement of mitochondrial functionality, and alleviation of local hypoxia associated with adipocyte hypertrophy (53). Furthermore, exercise-induced myokines, including IL-10 and irisin, further attenuate adipose inflammatory pathways (54). While exercise consistently reduces adipose inflammatory markers in humans with obesity, evidence for additive or synergistic cytokine modulation with vitamin D supplementation remains heterogeneous in clinical trials and is therefore best classified as mechanistically plausible rather than definitively established (55). This synergistic modulation may engender a more advantageous adipose microenvironment conducive to overarching metabolic enhancements. Aerobic exercise appears particularly effective in reducing visceral adiposity and adipose hypoxia, whereas resistance training contributes to inflammation reduction indirectly through improved muscle insulin sensitivity and myokine release, effects that may be amplified in the presence of adequate vitamin D status.

Emerging evidence from recent narrative reviews extends the concept of shared anti-inflammatory and antioxidant mechanisms between vitamin D and physical exercise into neuroinflammatory and neurodegenerative contexts, highlighting additional molecular convergence relevant to systemic regulation. Specifically, Farina and Crescioli (49) review preclinical and human neurodegeneration literature linking vitamin D–VDR axis activity with modulation of pro-inflammatory cytokines (e.g., TNFα, IL-1β, IL-6), neuroinflammatory signaling cascades such as NLRP3/caspase-1, and key antioxidant networks including Nrf2-dependent gene expression, all of which overlap with pathways modulated by exercise training in muscle and adipose tissues. These authors propose that combined vitamin D sufficiency and exercise may reinforce anti-inflammatory, antioxidant, and neurotrophic networks that preserve synaptic and cellular integrity in aging and disease, although they emphasize that definitive clinical evidence for synergistic effects remains to be established and that trial results are heterogeneous. This perspective reinforces the broader mechanistic theme that vitamin D and exercise converge on shared biomolecular targets influencing inflammatory homeostasis and tissue resilience across organ systems, thereby supporting a systemic interpretation of interaction between metabolic and immune pathways relevant to obesity and its comorbidities (49).

In addressing metabolic adaptation in overweight and obese populations, systematically demonstrates that vitamin D supplementation can amplify the transcriptomic responsiveness of skeletal muscle to exercise, particularly in pathways governing lipid metabolism and energy homeostasis (16). Integrated analysis of transcriptomic data revealed up-regulation of vitamin D response genes post-intervention, with enriched pathways in lipid digestion and absorption, while meta-analytic stratification of randomized trials showed significant reductions in waist circumference with combined vitamin D and exercise interventions. These findings align with and extend our discussion of adipokine modulation and insulin sensitivity by establishing putative molecular targets (e.g., lipid and carbohydrate metabolism gene sets) that are specifically responsive to vitamin D status in the context of exercise stimuli. Moreover, subgroup effects observed in older adults and aerobic exercise paradigms underscore the importance of population- and modality-specific interactions, suggesting that biochemical responsiveness to combined interventions may vary with phenotypic and physiological context (16).

Peng et al. (16) contribute uniquely by integrating transcriptomic analyses with meta-analytic evidence, thereby moving beyond biomarker-level observations. Their identification of vitamin D-responsive gene clusters involved in lipid absorption and mitochondrial energy metabolism provides molecular specificity that was previously lacking in clinical obesity trials. However, their meta-analytic findings demonstrate that anthropometric benefits (e.g., waist circumference reduction) are modest and more pronounced in aerobic exercise paradigms and older adults. When contrasted with earlier RCTs in this manuscript that showed largely neutral additive effects in metabolically heterogeneous cohorts, these findings suggest that vitamin D may function primarily as a permissive modulator of transcriptional responsiveness rather than a primary driver of weight reduction. This interpretation supports the conceptual framing of vitamin D as context-dependent and adjunctive rather than synergistically transformative (16).

### 3.2 Regulation of TLR4, FATP4, and adipokines in obesity

The inflammatory response associated with obesity is exacerbated by the activation of toll-like receptor 4 (TLR4), which detects saturated fatty acids and initiates downstream NF-κB signaling pathways. Vitamin D exerts a direct inhibitory effect on TLR4 expression and signaling within adipocytes and macrophages, thereby diminishing cytokine release and oxidative stress (56). Similarly, exercise contributes to the downregulation of TLR4 expression in adipose tissue, particularly when accompanied by reductions in circulating free fatty acids and enhancements in lipid oxidation (57). Furthermore, vitamin D influences lipid transport and metabolism through the regulation of fatty acid transport protein 4 (FATP4), which serves as a critical regulator of fatty acid uptake in adipocytes. Dysregulated FATP4 plays a pivotal role in the unwelcome accumulation of fat in non-adipose tissues and the ensuing lipotoxicity. The ability of Vitamin D to dampen FATP4 expression, combined with the boost in mitochondrial fatty acid oxidation from exercise, curtails adipocyte enlargement and fine-tunes lipid distribution (58, 59). Both of these strategies impact the release of adipokines, elevating adiponectin levels while mitigating leptin resistance in those battling obesity (60, 61). The enhancement of adipokine profiles fosters improved insulin sensitivity, diminishes inflammation, and promotes superior metabolic control. The powerful alliance of vitamin D and physical activity amplifies these positive transformations, shaping a more insulin-sensitive hormonal landscape.

### 3.3 Improvements in insulin signaling (IRS-1, GLUT4, AKT activation)

Vitamin D and physical activity intersect at numerous pivotal points along the insulin signaling pathway. Vitamin D amplifies the phosphorylation and function of insulin receptor substrate-1 (IRS-1), boosts the production of GLUT4, and promotes PI3K/Akt activation, thereby enhancing glucose uptake in both muscle and fat tissues (62). This impact is partially driven by VDR’s regulation of calcium balance and its transcriptional influence on insulin-sensitive genes. Exercise stimulates analogous pathways via AMPK and contraction-driven GLUT4 translocation, leading to a rise in glucose disposal even amidst insulin resistance (63). Ongoing aerobic and resistance training further revitalizes IRS-1 functionality, may enhance mitochondrial performance, and escalates Akt phosphorylation (64). Exercise-induced increases in GLUT4 expression and insulin sensitivity are empirically supported in human obesity cohorts. However, amplification of these adaptations by vitamin D sufficiency remains primarily mechanistically inferred, with limited direct confirmation in adequately powered obesity-specific trials (65). Collectively, these processes lower fasting glucose levels, decrease HOMA-IR, and improve overall insulin responsiveness more effectively than either approach alone. However, whether these mechanistic overlaps translate into additive or synergistic clinical benefit in humans with obesity remains unproven, as deficiency-stratified, adequately powered trials with standardized exercise dose and clinically meaningful endpoints are lacking.

### 3.4 Evidence from both animal and human studies to enhance insulin sensitivity and metabolic homeostasis

Insights into the complementary interactions between vitamin D and physical activity on insulin sensitivity and metabolic equilibrium are derived from a comprehensive amalgamation of animal models and human clinical investigations, each contributing unique mechanistic and translational insights. Importantly, many human studies referenced herein include heterogeneous populations (e.g., elderly individuals, patients with chronic disease, or vitamin D–deficient but subjects without obesity), and direct extrapolation of these findings to obesity-specific pathophysiology should be interpreted with caution. Animal research facilitates meticulous regulation of environmental, nutritional, and genetic variables, thereby allowing for an in-depth analysis of the molecular mechanisms through which vitamin D and physical activity might exert their influence on glucose homeostasis. Conversely, human investigations substantiate these findings within practical contexts, thereby affirming their clinical applicability for individuals with overweight or obesity.

An investigation aimed to elucidate the combined effects of vitamin D supplementation and aquatic exercise on obesity and hepatic steatosis induced by a high-fat diet (HFD) in a rat model, with particular emphasis on the regulatory mechanisms involving fatty acid transport protein-4 (FATP4) and Toll-like receptor-4 (TLR4). A total of thirty male rats were systematically allocated into five distinct groups, each subjected to varied combinations of HFD, vitamin D supplementation, and exercise regimens. The administration of HFD resulted in increased adiposity, heightened inflammatory responses, dysregulation of adipokines, and pathological alterations in liver tissue, which were concomitant with the upregulation of FATP4 and TLR4 in both adipose and hepatic tissues. In this study, the inclusion of vitamin D and physical exercise was associated with an enhancement in serum lipid profiles, a reduction in pro-inflammatory cytokines IL-6 and TNF-α, and a mitigation of histopathological liver damage. The integration of both interventions yielded the most pronounced decreases in the expression levels of FATP4 and TLR4, as well as in inflammatory markers. These results indicates that there might be a synergistic effect of vitamin D and exercise in counteracting obesity and steatosis, potentially through the modulation of lipid transport and inflammatory signaling pathways (66) (Table 1).

**TABLE 1:** Summary of evidence from animal and human studies on vitamin D–exercise interaction for improving insulin sensitivity and metabolic homeostasis.

Type of evidence	Study model	Population	Type of exercise	Dosage of vit D	Metabolic outcomes	Inflammatory outcomes	Insulin/ glycemic outcomes	Key findings	Quantitative outcomes	References
Preclinical	Rats (HFD-induced obesity)	Male rats; high-fat diet–induced obesity; insulin resistant	Swimming exercise	500 IU/kg	↓ body weight, ↓ hepatic steatosis, improved lipid profile	↓ TNF-α, ↓ IL-6, ↓ TLR4, ↓ FATP4	Improved adipokines	Combined Vit D + exercise strongly reduced inflammation and steatosis via FATP4/TLR4 downregulation.	Body weight: HFD group final ≈418.6 ± 31.7 g vs. combined ≈312 ± 13.4 g; Fat weight: HFD ≈20.6 ± 5.4 g vs. combined ≈9.7 ± 0.8 g (*p* < 0.01 vs. HFD)	(66)
	Mice with obesity (HFS diet)	Male mice; high-fat/high-sucrose diet–induced obesity; insulin resistant	Voluntary exercise	15,000 UI⋅kg^–1^	↓ adiposity (exercise), improved hepatic steatosis	↓ chemokines in adipose tissue	↑ insulin sensitivity (additive effect)	Combined Vit D + exercise had additive benefits on hepatic steatosis and insulin sensitivity.	Mean adipocyte size was 11% lower (*P* < 0.05) compared with the HFS group	(68)
	Ovariectomized rats	Ovariectomized female rats; postmenopausal obesity model; metabolic dysfunction	Aerobic training	9000 IU by injection and 1000 IU by food intake per week	↓ body weight, ↓ visceral fat, improved lipid profile	Not reported	↓ glucose, ↓ insulin, ↓ HOMA-IR	Combined Vit D + exercise significantly restored metabolic health.	OVX + AT + Vit D (*P* < 0.001); whereas these variables increased significantly in OVX + C (*P* < 0.001) and SHAM (*P* < 0.023) groups	(71)
	Western-diet in rats with obesity	Rats fed Western diet; diet-induced obesity with dyslipidemia and inflammation	Swimming	50000 IU/w	↓ adiposity (exercise), ↓ TG (more with Vit D)	↓ TNF-α, ↓ leptin	Improved glycemia	Exercise was the primary driver of metabolic and inflammatory improvements; Vit D added mild TG reduction.	–	(72)
	Rats with T2DM	Rats with type 2 diabetes mellitus; obesity and insulin resistant	Aerobic training	50000 IU/w	↓ BW, ↓ BMI, ↓ visceral fat	Not reported	↓ insulin, ↓ glucose, ↓ HOMA-IR	Combined aerobic + Vit D strongly upregulated AMPK, PGC-1α, UCP1 gene expression.	AT + Vit D significantly upregulated AMPK (*p* = 0.004; *p* = 0.001), PGC-1α (*p* = 0.010; *p* = 0.001), and UCP1 (*p* = 0.032; *p* = 0.001) gene expression, respectively. Also, AT induced more significant upregulations in the AMPK (*p* = 0.001), PGC-1α (*p* = 0.001), and UCP1 gene expression (*p* = 0.001) than Vit D	(75)
Clinical	Adults with overweight/ obesity	Adults with overweight and obesity; mixed sex; generally healthy but low-grade inflammation	Resistance training	4000 IU/day	No significant metabolic changes	No change in CRP, TNF-α, IL-6	No additional effect on insulin markers	Exercise improved TNF-α regulation, but Vit D did not enhance inflammatory responses.	Significant correlation between percent body fat and CRP at baseline (*r* = 0.45, *P* = 0.04), and between serum 25OHD and CRP at 12 weeks (*r* = 0.49, *P* = 0.03). The PL group had a significant increase in 25 μg/ml LPS + polymixin B-stimulated TNFα production (*P* = 0.04), and both groups had a significant reduction in unstimulated TNFα production (*P* < 0.05)	(67)
	Elderly women with T2D and Vit D deficiency	Elderly women; type 2 diabetes; vitamin D deficient; obesity	Circuit training	1,200 IU per day	↓ body fat (exercise), improved lipids	Not reported	↓ fasting glucose, ↓ fasting insulin, ↓ HOMA-IR (trend)	Exercise improved adiposity; Vit D enhanced training effects on metabolic health.	TG (*p* = 0.031) and LDL-C (*p* = 0.001) TC (*p* < 0.001) and HDL-C (*p* < 0.001)	(69)
	Adolescents with obesity and Vit D deficiency	Adolescents with obesity; vitamin D deficient; sedentary	Treadmill or jump rope	2,000 IU/day	↓ body fat, ↓ TG, ↓ TC, ↑ HDL	↓ leptin	Some reductions in insulin resistance	Exercise produced strong benefits; Vit D gave additional lipid improvements.	Significant decreases in leptin, body weight, BMI percentile, body fat percentage, triglycerides, total cholesterol, and LDL were found in the exercise groups (*p* < 0.05). HDL increased significantly in these groups (*p* < 0.05)	(70)
	Overweight women (30–35 y)	Overweight adult women (30–35 years); metabolically at risk	Aerobic exercise	–	↓ body weight, improved lipid profile	Not reported	Improved glucose homeostasis	Combined Vit D + aerobic exercise improved body composition and metabolic markers.	–	(73)
	Adults with overweight	Adults with overweight/obesity; non-diabetic; mixed sex	HIIT	100 μg⋅day^–1^	HIIT ↓ TG, ↓ glucose AUC	Not reported	HIIT improved glucose tolerance; Vit D attenuated benefits	Vit D did not enhance HIIT results; glucose tolerance improved mainly from HIIT.	A decrease in glucose (829 ± 110–786 ± 139 mmol⋅h^–1^⋅L^–1^; *P* = 0.043) and insulin (8101 ± 4755–7024 ± 4489 mU⋅h^–1^⋅L^–1^; *P* = 0.049) area under the curve (AUC). Supplementation increased 25-hydroxyvitamin D_3_ concentration by 120% to a sufficiency status (*P* < 0.001).	(74)
	Patients with T2DM	Adults with type 2 diabetes mellitus; overweight/obesity; mixed sex	Aerobic training	50000 IU/w	Improved lipid profile; ↓ TC, ↓ TG, ↓ LDL	↓ IL-6, ↓ IFN-γ, ↓ CXCL13, ↓ TGF-β1	Improved glycemic markers	Combined Vit D + AT enhanced anti-inflammatory gene expression.	AT + Vit D significantly downregulated IL-6 (*P* = 0.013; *P* = 0.025), IL-10 (*P* = 0.012; *P* = 0.026), CD27 (*P* = 0.023; *P* = 0.041), CXCL13 (*P* = 0.014; *P* = 0.025), IFN-γ (*P* = 0.017; *P* = 0.026), and TGF-β1 (*P* = 0.001; *P* = 0.028)	(76)
	Women with obesity	Women with obesity; cardiometabolic risk factors	Aerobic training	50,000 IU/wk	Improved adiposity and lipid profile	Not reported	Improved insulin resistance	Vit D + exercise showed greater cardiometabolic improvements than exercise alone.	Vitamin D supplementation increased serum 25-hydroxyvitamin D levels significantly (*P* < 0.05). Aerobic training caused significant improvement in body weight, waist circumference, HDL-cholesterol, blood glucose and insulin resistance index (*P* < 0.05). Vitamin D and aerobic training lead to greater improvement in adiposity indices, HDL-cholesterol, LDL-cholesterol, total cholesterol, triglyceride, blood glucose and insulin resistance index (*P* < 0.05).	(77)
	Elderly women with NAFLD and Vit D deficiency	Elderly women with obesity; NAFLD and vitamin D deficiency	Aerobic training	50,000 IU/wk	↓ liver enzymes, ↓ fat mass	Not reported	Improved glycemic indices	Combination therapy most effectively reduced NAFLD grade and improved metabolic profiles.	–	(78)

↓ Reduction; ↑ Increase. HFD, high-fat diet; Vit D, vitamin D; CRP, C-reactive protein; TNF-α, tumor necrosis factor-alpha; IL-6, interleukin-6; TLR4, toll-like receptor 4; FATP4, fatty acid transport protein 4; TG, triglycerides; TC, total cholesterol; HDL, high-density lipoprotein; LDL, low-density lipoprotein; HFS, high-fat high-sucrose diet; T2D, type 2 diabetes; T2DM, type 2 diabetes mellitus; AUC, area under the curve; HIIT, high-intensity interval training; BW, body weight; BMI, body mass index; PGC-1α, peroxisome proliferator-activated receptor-γ coactivator-1α; AMPK, AMP-activated protein kinase; UCP1, uncoupling protein-1; IFN-γ, interferon-gamma; CXCL13, C-X-C motif chemokine ligand 13; TGF-β1, transforming growth factor-beta 1; NAFLD, non-alcoholic fatty liver disease.

This study by Kolieb et al. suggests several methodological strengths, including a controlled experimental design with multiple intervention arms (vitamin D, swimming exercise, and their combination), baseline equivalence between groups, and the assessment of both physiological outcomes and mechanistic biomarkers such as FATP4 and TLR4 expression. These features enhance internal validity and allow exploratory investigation of potential pathways underlying metabolic improvements in a high-fat diet rat model. However, important statistical limitations should be considered when interpreting the findings. The small sample size (*n* = 6 per group) reduces statistical power and increases susceptibility to random variation and inflated effect sizes. Additionally, the large number of outcome measures raises concerns about multiple comparisons and potential false-positive results, particularly in the absence of clearly reported correction procedures or power calculations. While the observed improvements in metabolic and inflammatory markers are biologically plausible and consistent with existing preclinical literature, the mechanistic conclusions regarding FATP4 and TLR4 may be overstated, as the study suggests associations rather than causal pathway confirmation. Furthermore, the use of a controlled animal model limits external validity and generalizability to human populations. Overall, the findings should be interpreted as exploratory and hypothesis-generating, warranting replication in larger and more rigorously powered studies before strong conclusions can be drawn.

Another study aimed to assess the impact of vitamin D supplementation on exercise-induced alterations in inflammatory biomarkers among subjects with overweight and obesity participating in a 12-weeks progressive resistance training program. A cohort of twenty-three participants was assigned to receive either 4,000 IU/day of vitamin D or a placebo. The resistance training regimen alone resulted in a reduction of unstimulated TNF-α production, whereas the placebo group exhibited an elevation in LPS-stimulated TNF-α levels. Nevertheless, vitamin D supplementation did not produce significant changes in CRP, IL-6, TNF-α, or ALT when compared to the placebo. Correlational analyses revealed that increased body fat was related to elevated CRP levels, while heightened serum 25OHD levels were associated with decreased CRP following the intervention. In summary, vitamin D supplementation elevated serum 25OHD levels but did not enhance exercise-induced anti-inflammatory responses. The findings imply that resistance training effectively improves inflammatory markers independent of vitamin D supplementation within this demographic (67). This study presents several methodological strengths, including a human-based experimental design involving subjects with overweight and obesity undergoing structured exercise training, which may enhance external validity compared with animal studies. The investigation used a controlled supplementation protocol and assessed relevant inflammatory biomarkers, allowing evaluation of whether vitamin D provides additional anti-inflammatory benefits beyond exercise alone. However, some statistical and methodological considerations should be noted. Sample size and statistical power may limit the ability to detect small or moderate effects, particularly given the variability inherent in inflammatory markers. Additionally, factors such as baseline vitamin D status, individual responsiveness to supplementation, adherence variability, and potential confounders related to lifestyle or metabolic heterogeneity could influence outcomes. The absence of significant changes in inflammatory biomarkers suggests that vitamin D supplementation did not confer additive benefits under the study conditions, but null findings should be interpreted cautiously, as they may reflect insufficient dosing, duration, or statistical power rather than true lack of effect. Overall, the results appear methodologically sound and reasonably reliable within the study context, yet further research with larger samples and stratified analyses is warranted to clarify whether specific subgroups may benefit from combined vitamin D supplementation and exercise interventions.

The aim of another study was to evaluate the extent to which the integration of voluntary physical exercise (PE) with vitamin D (VD) supplementation contributes to the enhancement of metabolic health in murine models subjected to a high-fat/sucrose dietary regimen. Following the induction of obesity through dietary means, the mice were subjected to a regimen of 15 weeks of PE, VD, or a combination of both treatments. The implementation of PE resulted in a significant reduction in body mass, adiposity, and the hypertrophy of adipocytes, whereas VD administration in isolation exhibited negligible effects on the overall body composition. Both interventions led to a decrease in inflammation within adipose tissue; however, the combined application of PE and VD yielded cumulative advantages in terms of insulin sensitivity and hepatic steatosis. Histological examination of liver tissues demonstrated a reduction in both the size and quantity of lipid droplets, while gene expression analysis indicated a downregulation of enzymes associated with *de novo* lipogenesis. These data demonstrated that vitamin D might be able to augment the exercise-induced enhancements in hepatic metabolic function and insulin responsiveness, thereby endorsing the adoption of integrated approaches for the management of obesity-related metabolic disorders (68). The study by Marziou et al. (68) employs a controlled experimental design in diet-induced mice with obesity, examining the independent and combined effects of voluntary exercise and vitamin D supplementation, which strengthens internal validity and allows assessment of potential synergistic benefits. The reported metabolic improvements are biologically plausible and align with existing preclinical evidence. However, relatively small sample sizes and multiple outcome measures may limit statistical power and increase the risk of type I error if multiple comparisons were not adequately controlled. Additionally, mechanistic interpretations should be made cautiously, as associations do not confirm causality. As an animal study, external validity is limited, and findings should be considered exploratory pending confirmation in well-powered human trials.

Another study analyzed the impact of vitamin D supplementation in conjunction with circuit training on obesity-related parameters among elderly women with type 2 diabetes and exhibiting vitamin D deficiency. A total of fifty-two subjects were allocated to one of four groups: vitamin D plus training (D + T), training exclusively (T), vitamin D solely (D), or a control group. Throughout the course of 12 weeks, the training regimen yielded significant reductions in body weight, fat mass, and visceral fat, as well as enhancements in lipid markers, whereas the vitamin D only group demonstrated negligible alterations. The D + T group exhibited favorable trends in fasting glucose, insulin levels, and HOMA-IR, although these effects did not achieve statistical superiority over the training alone group. Notably, lean mass did not exhibit any significant changes across the various groups. The results shown that circuit training serves as the principal catalyst for enhancements in adiposity and lipid metabolism, while vitamin D may provide a modest augmentation of insulin-related outcomes when combined with physical exercise (69). The study by Kim et al. investigates the combined effects of vitamin D supplementation and circuit training on obesity indices and insulin resistance in vitamin D–deficient elderly women with type 2 diabetes, providing clinically relevant human data that may enhance external validity. The intervention-based design allows evaluation of potential additive benefits; however, limitations such as relatively small sample size, possible selection bias, and variability in individual responses may affect statistical power and generalizability. Additionally, lifestyle and metabolic heterogeneity among participants could introduce confounding factors. While the reported improvements are promising and biologically plausible, the findings should be interpreted cautiously, and larger, well-controlled trials are needed to confirm the reliability and broader applicability of the results.

Another randomized study investigated the effects of treadmill running (R) and jump rope (JR) training, with the inclusion or exclusion of vitamin D supplementation, in sedentary adolescents exhibiting obesity and vitamin D deficiency. Over the duration of 8 weeks, both exercise modalities, irrespective of vitamin D status, demonstrated significant reductions in leptin levels, body fat percentage, overall weight, triglyceride levels, total cholesterol, and low-density lipoprotein (LDL), alongside an increase in high-density lipoprotein (HDL). The administration of vitamin D in isolation exhibited a negligible metabolic influence when juxtaposed with the exercise interventions. No statistically significant disparities were observed between the R + VitD and JR + VitD cohorts, suggesting an equivalent efficacy of both exercise modalities. Although all exercise groups demonstrated superior outcomes compared to the vitamin D-only and control groups, the addition of vitamin D did not produce outcomes surpassing those attributable to exercise alone. These data endorsed aerobic and plyometric training as efficacious interventions for enhancing adiposity and lipid profiles in adolescents with obesity (70). The study by Sheikholeslami-Vatani et al. (70) examines the effects of different exercise programs combined with vitamin D supplementation on lipid profile and leptin levels in sedentary, adolescents with obesity and vitamin D deficiency, offering clinically relevant insights within a human population. The intervention-based design supports evaluation of potential combined effects; however, statistical considerations such as sample size, potential variability in adherence, and heterogeneity in adolescent growth and metabolic responses may influence the robustness of the findings. Additionally, multiple outcome measures may increase the risk of type I error if not adequately controlled. While the reported improvements are biologically plausible, the results should be interpreted cautiously and confirmed through larger, well-powered randomized studies.

The goal of another study was to explore the combined effect of aerobic exercise and vitamin D supplementation in ovariectomized rats, which serve as a pertinent model for postmenopausal obesity. The experimental subjects were subjected to aerobic training, vitamin D administration, a combination of both modalities, or were designated as control subjects. The cohort receiving the combined aerobic training and vitamin D treatment (OVX + AT + VitD) demonstrated statistically significant reductions in body weight, visceral fat accumulation, body mass index (BMI), and food consumption in comparison to the control group. Markers indicative of lipid profiles (total cholesterol, triglycerides, high-density lipoprotein cholesterol, low-density lipoprotein cholesterol), alongside glucose levels, insulin concentrations, and homeostasis model assessment of insulin resistance (HOMA-IR) exhibited improvements across the various intervention groups, with the most pronounced enhancements evident in the combined treatment group. The administration of vitamin D in isolation yielded moderate beneficial effects. The results imply that the energy expenditure induced by exercise and the consequent activation of muscle may work complementary with vitamin D to augment insulin sensitivity and maintain metabolic equilibrium, thereby endorsing the efficacy of combined therapeutic strategies for addressing metabolic risks associated with postmenopausal conditions (71). The study by Babaei et al. (71) investigates the interaction between aerobic exercise training and vitamin D supplementation on lipid profiles and insulin resistance in ovariectomized rats, using a controlled experimental design that supports internal validity and exploration of potential synergistic effects. The findings suggest beneficial metabolic changes; however, small sample sizes typical of animal studies may limit statistical power and increase variability. Additionally, mechanistic interpretations should be made cautiously, as observed associations do not confirm causality. As an animal model, external validity is limited, and extrapolation to human populations requires confirmation through well-designed clinical trials.

Another work assessed the interaction between swimming exercise training (ET) and vitamin D supplementation in their effects on mitigating obesity induced by a Western diet (WD) in a rat model. Rats subjected to a WD diet and leading a sedentary lifestyle exhibited the development of obesity, hypercholesterolemia, hyperleptinemia, and an increase in TNF-α levels. The implementation of swimming ET might result in a significant reduction in abdominal adiposity, leptin levels, TNF-α, alongside enhancements in glycemic control and lipid profiles. Notably, vitamin D supplementation, when administered in isolation, failed to prevent adiposity and did not substantially augment the majority of the metabolic improvements induced by exercise; however, it did exert a modest potentiation of the reduction in triglyceride levels. The primary anti-obesity effects were predominantly attributed to exercise, with vitamin D contributing only marginally to the overall benefit. These findings exhibited that regular aerobic training serves as a more effective modulator of adiposity and inflammatory responses compared to vitamin D in animals fed a WD, thereby underscoring the role of exercise as the principal protective factor in the management of obesity (72). These findings suggest that exercise may attenuate obesity-related outcomes independently of vitamin D supplementation, highlighting the dominant role of physical activity in this context. However, statistical limitations such as small group sizes and multiple measured outcomes may affect power and increase the risk of type I error if not adequately controlled. Additionally, as an animal study, external validity is limited, and translation of these findings to human populations should be approached cautiously pending confirmation in clinical trials.

Hassan et al. (73) investigated the impact of vitamin D supplementation in conjunction with an aerobic exercise regimen on biochemical markers in overweight females aged 30–35 years. Over a duration of 12 weeks, participants who received vitamin D alongside a structured aerobic training program demonstrated significant reductions in body weight, total body fat, cholesterol, triglycerides, and LDL-C, coupled with increases in HDL-C and serum 25OHD levels. The intervention markedly enhanced overall metabolic health and facilitated weight loss more effectively than pre-existing lifestyle habits. Vitamin D augmented the advantageous effects of exercise on lipid metabolism and vitamin D levels, thereby contributing to improved physiological functionality. The results suggest the importance of incorporating vitamin D supplementation within aerobic training protocols to optimize metabolic profiles and foster effective weight management in overweight individuals (73). This study is providing clinically relevant human data that enhance external validity. The intervention-based design allows assessment of potential additive benefits on weight-related metabolic variables. However, statistical considerations such as sample size, variability in adherence, and heterogeneity in participants’ baseline metabolic status may limit statistical power and the robustness of conclusions. Additionally, multiple biochemical outcomes increase the risk of type I error if not properly adjusted. While the reported improvements are biologically plausible, the findings should be interpreted cautiously, and confirmation through larger, well-controlled trials is warranted.

In another study, Lithgow et al. (74) investigated the extent to which vitamin D supplementation may amplify the metabolic consequences of high-intensity intermittent training (HIIT) on glycemic regulation among individuals with overweight and obesity. A total of twenty subjects underwent a 6-weeks HIIT regimen and were assigned randomly to receive either vitamin D3 (100 μg/day) or a placebo. The HIIT protocol resulted in significant enhancements in glucose tolerance, a decrease in the area-under-the-curve values for glucose and insulin, as well as a reduction in triglyceride levels. Although vitamin D supplementation led to a noteworthy increase in serum 25OHD concentrations, it did not significantly improve insulin sensitivity or glycemic parameters. Interestingly, vitamin D appeared to slightly mitigate the reduction in glucose levels during the oral glucose tolerance test (OGTT), although this effect was not deemed clinically significant. In conclusion, while HIIT alone yielded substantial metabolic advantages, the addition of vitamin D did not result in supplementary improvements. These results indicate that HIIT is potentially efficacious for glycemic management irrespective of vitamin D supplementation in non-diabetic populations (74). The intervention design of this study allows evaluation of potential additive effects on glucose metabolism. However, limitations such as small sample size, variability in participant adherence, and individual metabolic heterogeneity may reduce statistical power and the reliability of conclusions. Additionally, multiple outcomes may increase the risk of type I error if not properly controlled. While the reported improvements in glycemic measures are biologically plausible, the results should be interpreted cautiously, and further well-powered trials are needed to confirm these findings.

An investigation examined the impact of aerobic training (AT) and vitamin D supplementation on adiposity, glycemic parameters, and the expression of visceral adipose tissue genes in a model of type 2 diabetes using rats. The application of AT, vitamin D, and the combination of AT and vitamin D collectively resulted in reductions in body weight, body mass index (BMI), waist circumference, visceral fat accumulation, glucose levels, insulin concentrations, and homeostasis model assessment of insulin resistance (HOMA-IR) when compared to diabetic control groups. All treatment modalities significantly upregulated the expression of AMPK, PGC-1α, and UCP-1, with the group receiving the combined AT and vitamin D intervention exhibiting the most pronounced gene induction. Aerobic training elicited more substantial molecular effects than vitamin D supplementation in isolation; however, the latter enhanced the exercise-induced improvements. These data suggest potential significant interactions between vitamin D and aerobic training in promoting mitochondrial biogenesis, thermogenic gene expression, and the maintenance of metabolic homeostasis within diabetic adipose tissue (75). However, small sample sizes and multiple measured outcomes may limit statistical power and increase the risk of type I error if adjustments for multiple comparisons are not applied. Additionally, mechanistic interpretations should be made cautiously, as observed associations do not establish causality. As an animal study, external validity is limited, and extrapolation to human populations requires confirmation through well-designed clinical trials.

Another study evaluated the impact of aerobic training (AT) and vitamin D supplementation on lipid profiles, hepatic enzyme levels, and the expression of inflammatory genes in males with type 2 diabetes mellitus. Following an 8-weeks intervention period, both AT, vitamin D, and their combination (AT + VitD) resulted in reductions of total cholesterol (TC), triglycerides (TG), low-density lipoprotein (LDL), aspartate aminotransferase (AST), alanine aminotransferase (ALT), and gamma-glutamyl transferase (GGT), alongside an elevation in high-density lipoprotein (HDL), with the combinatorial treatment exhibiting the most pronounced enhancements. Markers of inflammatory gene expression within peripheral blood mononuclear cells (PBMCs), such as interleukin-6 (IL-6), interleukin-10 (IL-10), CD27, C-X-C motif chemokine 13 (CXCL13), interferon-gamma (IFN-γ), and transforming growth factor-beta 1 (TGF-β1), experienced significant downregulation across all intervention cohorts, with the AT + VitD group demonstrating the most substantial reduction. Conversely, the control cohort exhibited a deterioration in inflammatory profiles. The results suggest that vitamin D may augment the anti-inflammatory and metabolic advantages associated with aerobic training, thereby endorsing the efficacy of combined therapeutic strategies for the management of T2DM (76).

The aim of another study was to assess the interaction between swimming exercise training (ET) and vitamin D supplementation in their effects on mitigating obesity induced by a Western diet (WD) in a rat model. Rats subjected to a WD diet and leading a sedentary lifestyle exhibited the development of obesity, hypercholesterolemia, hyperleptinemia, and an increase in TNF-α levels. The implementation of swimming ET resulted in a significant reduction in abdominal adiposity, leptin levels, TNF-α, alongside enhancements in glycemic control and lipid profiles. Notably, vitamin D supplementation, when administered in isolation, failed to prevent adiposity and did not substantially augment the majority of the metabolic improvements induced by exercise; however, it did exert a modest potentiation of the reduction in triglyceride levels. The primary anti-obesity effects were predominantly attributed to exercise, with vitamin D contributing only marginally to the overall benefit. These data indicated that regular aerobic training serves as a more effective modulator of adiposity and inflammatory responses compared to vitamin D in animals fed a WD, thereby underscoring the role of exercise as the principal protective factor in the management of obesity (77).

Another controlled trial examined the impact of aerobic training (AT) in conjunction with vitamin D supplementation on elderly women diagnosed with non-alcoholic fatty liver disease (NAFLD) and concomitant vitamin D deficiency. Upon completion of an 8-weeks intervention, there was a statistically significant enhancement in liver fat grading within the AT + VitD, AT, and VitD cohorts, with the combination treatment demonstrating the most pronounced reduction of 60%. Furthermore, the AT + VitD group exhibited superior advancements in liver enzyme levels, anthropometric parameters, glycemic control metrics, and lipid profiles. Notably, vitamin D concentrations displayed an inverse correlation with the severity of fatty liver across all groups analyzed. In contrast, the control group experienced a deterioration in NAFLD. The results underscore the premise that adequate vitamin D levels potentiate the metabolic and hepatic advantages of aerobic training, thereby highlighting the critical need to address vitamin D deficiency to optimize exercise efficacy in the management of NAFLD (78). Based on the empirical evidence presented, it may be inferred that the administration of vitamin D supplementation, in conjunction with diverse modalities of physical exercise, including aerobic activities, resistance training, circuit workouts, swimming, and high-intensity interval training, consistently may enhance metabolic health parameters. The observed advantages encompass reductions in body mass, adiposity, body mass index (BMI), visceral and abdominal fat deposits, lipid profiles (triglycerides, total cholesterol, low-density lipoprotein), and inflammatory biomarkers (C-reactive protein, tumor necrosis factor-alpha, interleukin-6), alongside enhancements in insulin sensitivity, glucose homeostasis, hepatic steatosis, and the expression of genes pertinent to adipose tissue metabolism (AMP-activated protein kinase, peroxisome proliferator-activated receptor gamma coactivator 1-alpha, uncoupling protein 1). This intervention allows evaluation of potential combined effects on metabolic and liver-related outcomes. However, limitations such as small sample size, variability in adherence, and heterogeneity in baseline disease severity may reduce statistical power and limit the robustness of conclusions. Additionally, multiple outcome measures increase the risk of type I error if not properly adjusted. While the reported improvements are biologically plausible, the findings should be interpreted cautiously, and confirmation through larger, well-controlled trials is warranted.

Across the studies reviewed, there are 11 human studies and 8 animal studies. While these studies consistently suggest that exercise improves metabolic, inflammatory, and cardiovascular outcomes, the additional benefit of vitamin D supplementation is less consistent, particularly in human trials, where sample sizes are often small, outcomes are heterogeneous, and statistical power is limited. Animal studies generally show synergistic effects of exercise and vitamin D, but translation to humans remains uncertain. Overall, the current evidence is insufficient to recommend routine combined vitamin D supplementation and exercise for metabolic improvement; exercise alone appears clearly beneficial, whereas vitamin D may provide additional effects only in specific populations (e.g., deficient individuals), and larger, well-powered human trials are needed to confirm any synergistic benefit. A systematic comparison of intervention duration across studies reveals important distinctions between short-term and long-term responses to combined vitamin D supplementation and exercise. Short-duration trials (typically 8–16 weeks) consistently demonstrate improvements in metabolic and functional outcomes driven primarily by exercise, including reductions in adiposity, enhanced insulin sensitivity, and improved physical performance; however, the incremental contribution of vitamin D supplementation during these shorter interventions appears modest and often limited to specific endpoints such as central adiposity, lower-body strength, or biochemical markers rather than broad clinical outcomes. These findings suggest that early adaptations are largely attributable to exercise-induced physiological changes, while vitamin D may function primarily as a permissive or supportive factor, particularly in individuals with baseline deficiency. In contrast, longer-term studies and extended observational analyses indicate that sustained exposure to adequate vitamin D status combined with habitual physical activity may influence broader trajectories related to biological aging, systemic inflammation, and functional reserve, although randomized clinical trials with multi-year follow-up have reported largely neutral results in generally healthy populations. The divergence between short- and long-term findings likely reflects differences in physiological targets and population characteristics: short-term interventions capture rapid metabolic adaptations, whereas longer durations may be required to observe structural or neurovascular changes, yet these effects may be attenuated in cohorts with high baseline health status or sufficient nutrient levels. Collectively, the evidence suggests that intervention duration interacts with baseline phenotype and outcome selection, emphasizing the need for longer, adequately powered trials that include stratification by vitamin D status and metabolic risk to determine whether combined interventions produce sustained synergistic benefits beyond exercise alone. Within the evidence-classification framework introduced in Section “2 Molecular and physiological basis of the vitamin D–exercise interaction,” several components of the adipose–insulin regulatory axis can be categorized with differing levels of certainty. Exercise-induced reductions in visceral adiposity, improvements in insulin sensitivity, and attenuation of systemic inflammatory markers (e.g., TNF-α, IL-6, CRP) are empirically supported in human populations with overweight and obesity. Improvements in GLUT4 translocation, AMPK activation, and mitochondrial biogenesis in response to exercise are also supported by human studies in obesity, although detailed intracellular signaling confirmation is more often derived from muscle biopsy substudies.

In contrast, direct vitamin D–mediated modulation of TLR4, FATP4, NF-κB signaling, and macrophage polarization within adipose tissue in obesity-specific human cohorts remains limited and heterogeneous. These interactions are therefore best categorized as mechanistically inferred, based primarily on animal models and cellular investigations. Evidence for coordinated or synergistic regulation of AMPK, PI3K/Akt, and adipokine signaling through combined vitamin D and exercise interventions in obesity-specific humans remains insufficient; such interactions are therefore currently classified as mechanistically inferred, despite biological plausibility and supportive preclinical findings. Collectively, within the adipose–insulin domain, exercise represents the empirically validated driver of metabolic improvement in obesity, whereas vitamin D functions as a context-dependent modulator whose additive effects remain to be definitively established in adequately powered human trials.

## 4 Effects on muscle metabolism and functional adaptation

Vitamin D and physical activity may interact together on skeletal muscle to enhance metabolic functionality, structural robustness, and comprehensive contractile efficacy in individuals with overweight or obesity. Vitamin D manifests direct influences on myocytes via the VDR, which is present in both type I and type II muscle fibers, consequently affecting pathways associated with muscular strength, mitochondrial metabolism, and calcium equilibrium. In populations with obesity, exercise-induced improvements in muscle strength, mitochondrial function, and glucose utilization are consistently supported by human evidence. Vitamin D–related influences on muscle physiology, including modulation of calcium handling, mitochondrial signaling, and anabolic pathways, are supported primarily by mechanistic and deficiency-focused studies, with limited direct confirmation of additive functional effects during exercise training in obesity. Consequently, vitamin D–mediated amplification of exercise-induced muscle remodeling should be interpreted as a context-dependent and partially inferred mechanism pending confirmation in adequately powered clinical trials. Exercise-induced improvements in skeletal muscle mitochondrial function, glucose uptake, and contractile performance are supported by robust human evidence in obesity and are therefore depicted as validated interactions in the axis model.

Jawed et al. (17) provides a narrative exploration of how vitamin D and physical activity act as co-modifiers of muscle health, emphasizing that vitamin D’s regulation of calcium homeostasis, protein synthesis, and muscle fiber function intersects with the mechanotransductive and metabolic effects of exercise. This synthesis supports the proposition that vitamin D sufficiency enhances the anabolic responses to exercise by facilitating calcium-dependent signaling and VDR-mediated transcriptional programs that promote muscle contractility and recovery. Importantly, Jawed et al. foreground the synergistic potential of combined interventions to optimize outcomes in strength and functional performance, thereby providing context for clinical observations of improved muscle quality with adequate vitamin D status in physically active cohorts. These mechanistic insights complement studies showing that combined exercise and vitamin D interventions are associated with enhanced muscle strength and functional outcomes, particularly when baseline deficiency is corrected. Together, these lines of evidence help bridge molecular and physiological scales, reinforcing the central role of vitamin D in shaping muscle adaptation alongside physical activity (17).

To maintain consistency with the evidence-classification framework introduced in Section “2 Molecular and physiological basis of the vitamin D–exercise interaction,” mechanistic interactions discussed in this section are explicitly identified as (i) empirically supported in humans with obesity, (ii) supported in related human contexts, or (iii) mechanistically inferred from animal, cellular, or indirect physiological evidence.

### 4.1 The role of vitamin D in muscle strength, mitochondrial metabolism, and calcium homeostasis

Vitamin D is critically involved in the modulation of intracellular calcium dynamics, which is a fundamental factor influencing the efficacy of muscle contractions. The interaction of 1,25-dihydroxyvitamin D with the VDR influences calcium absorption through L-type calcium channels and optimizes sarcoplasmic reticulum performance, thereby facilitating improved excitation–contraction coupling (27, 79). Vitamin D has been shown in cellular and animal models to promote mitochondrial biogenesis through AMPK and PGC-1α activation; however, direct evidence demonstrating clinically meaningful enhancement of mitochondrial adaptation during exercise in humans with obesity remains limited, which collectively augment ATP production while mitigating the accumulation of reactive oxygen species (ROS), pathways that are frequently compromised in muscle dysfunction associated with obesity (80). A deficiency in vitamin D status has been persistently linked to diminished muscle strength, fiber atrophy, and impaired mitochondrial function, thereby underscoring its significance in the preservation of muscle integrity and metabolic adaptability (81).

### 4.2 Exercise-induced muscle remodeling enhanced by vitamin D

Exercise catalyzes significant alterations in skeletal muscle architecture through mechanisms such as mechanical loading, calcium signaling, and metabolic stress, thereby activating cascades that govern hypertrophic responses, mitochondrial proliferation, and protein biosynthesis. Vitamin D may potentiate this adaptive remodeling by enhancing critical molecular regulators. The co-activation of the VDR in conjunction with endurance or resistance training elevates the expression levels of PGC-1α, thereby facilitating mitochondrial biogenesis and enhancing oxidative capacity (82). Simultaneously, Vitamin D–mediated enhancement of mTOR signaling and muscle hypertrophy is supported in mechanistic and deficiency-focused studies, but additive hypertrophic effects during resistance training in obesity populations remain mechanistically inferred (83). Crucially, vitamin D has been demonstrated to inhibit myostatin, a recognized negative regulator of muscle mass, thus amplifying exercise-induced anabolic responses and promoting enhanced muscle regeneration and remodeling (84). Collectively, these signaling pathways might foster a more robust adaptive response to exercise in individuals with overweight or obesity, who frequently exhibit diminished muscle plasticity as a consequence of chronic inflammation and metabolic inflexibility.

### 4.3 Improved glucose tolerance, protein synthesis, and muscle contractility

The potential synergistic effects of vitamin D and physical exercise may augment the metabolic health of skeletal muscle. Vitamin D may enhance insulin sensitivity by elevating the expression of GLUT4 in muscle cells and mitigating the accumulation of intramuscular lipids, two pivotal determinants associated with glucose intolerance in individuals with obesity (85). Exercise robustly induces GLUT4 translocation and enhances muscle glucose uptake in humans with obesity. Whether vitamin D supplementation produces cumulative or synergistic augmentation of this response remains uncertain and is not consistently demonstrated in clinical trials, culminating in enhanced whole-body glucose tolerance (86). Moreover, the integrated intervention fosters increased protein synthesis via mTOR activation and diminishes muscle fatigability by optimizing mitochondrial ATP production. Enhanced calcium homeostasis and excitation–contraction coupling further augment muscle contractility and overall performance (87). In summary, vitamin D and exercise operate through complementary mechanisms to facilitate functional muscle adaptations, enhance metabolic efficiency, and improve overall neuromuscular health in populations characterized by overweight and obesity. By contrast, vitamin D–mediated amplification of hypertrophic signaling and exercise responsiveness in obesity remains incompletely established in human trials and is therefore classified as mechanistically inferred within the current framework.

### 4.4 Evidence suggesting a potential additive interaction on muscle metabolism and function

An expanding body of evidence derived from both animal research and human clinical trials indicates that the concomitant administration of vitamin D and exercise might show enhancements in muscle metabolism, structural integrity, and functional performance, effects that exceed the outcomes of each intervention in isolation. These results emphasize the clinical significance of the vitamin D–exercise interplay in populations characterized by overweight and obesity, who often present with muscle weakness, mitochondrial dysfunction, and compromised metabolic flexibility. Although emerging preclinical and clinical findings suggest a potential interactive effect, the proposed vitamin D–exercise synergy should currently be regarded as a mechanistic hypothesis requiring further direct experimental validation. A study elucidated that the integration of aquatic exercise with vitamin D3 supplementation might yield significant enhanced advancements in bone metabolism in postmenopausal women with overweight or obesity experiencing vitamin D insufficiency (88). The implementation of aquatic training was associated with an augmentation in femoral bone mineral density (BMD), and its efficacy was further potentiated when combined with vitamin D, suggesting a protentional combined effect on the processes of bone remodeling. Vitamin D supplementation resulted in a notable elevation of serum 25(OH)D concentrations, whereas aquatic exercise in isolation did not modify vitamin D status. This combined intervention also resulted in the most substantial decrease in parathyroid hormone (PTH) levels, indicative of an improved calcium equilibrium and endocrine regulation concerning bone turnover. While vitamin D supplementation alone exhibited minimal influence on BMD, aquatic exercise independently fostered advantageous responses in bone metabolism. In summary, interventions that incorporate both aquatic exercise and vitamin D supplementation exhibited the most pronounced enhancements in markers indicative of bone health (88) (Table 2). This intervention is biologically plausible and the randomized design supports causal interpretation, but from a statistical perspective the sample size appears modest, which limits power and increases uncertainty around effect estimates, particularly if several bone markers were analyzed simultaneously. The analyses rely mainly on pre and post intervention and between group comparisons with limited multivariable adjustment, leaving room for residual confounding from factors such as baseline bone status, dietary calcium intake, or sunlight exposure. Details on allocation concealment and handling of missing data are limited, making it difficult to fully assess risk of bias. Overall, the findings are consistent with current understanding of bone metabolism in postmenopausal obesity and likely reliable in terms of direction of effect, but the magnitude and clinical significance of the results should be interpreted cautiously, as the statistical evidence is supportive rather than conclusive.

**TABLE 2:** Effects of vitamin D supplementation and exercise interventions on muscle and bone metabolism, functional performance, and metabolic health in subjects with overweight and obesity.

Model	Population	Intervention	Vitamin D dosage	Duration	Main effects	Quantitative outcomes	References
Animal	24-weeks-old male p62-deficient mice (obesity)	Control, Vitamin D (VD), Resistance training (RT), VD + RT	1000 IU vitamin D3/kg/d	10 weeks	Serum VitD ↑ in VD/VRT; fat mass ↑ only in control; skeletal muscle function preserved in VD; glucose ↓ and spleen mass ↓ in RT/VRT	Serum 25(OH)D: ↑ in VD and VRT vs. control (*P* < 0.05); Glucose: reduced in RT and VRT vs. control (*P* < 0.05); Fat mass: increased only in control (*P* < 0.05)	(90)
Human	40 postmenopausal women with obesity and vitamin D insufficiency	4 groups: Aquatic training + Vitamin D3 (ATD), Aquatic training + placebo (AT), Vitamin D3 only (D), Control (CON)	4000 IU/d	8 weeks	Femur BMD ↑ in ATD > AT > D/CON; Serum 25(OH)D ↑ in ATD and D; PTH ↓ in ATD > AT/D > CON	BMD: ATD > AT/D/CON	(88)
Human	50 older adults with overweight/obesity with vitamin D deficiency	Vitamin D3 (4000 IU/day) vs. placebo, all did multi-modal exercise from week 12–24	4000 IU/day	24 weeks	Serum 25(OH)D ↑; stair climb ↓; waist circumference and WHR ↓ with vitamin D + exercise; gait speed unaffected	The significant decrease in waist circumference (net difference: −4.4 cm [95%CI −8.1, −0.8 cm], *P* = 0.017) and WHR (net difference: −0.1 [95%CI −0.1, −0.02], *P* = 0.001)	(89)
Human	23 adults with overweight/obesity (age 26.1 ± 4.7 years) undergoing resistance training	Vitamin D3 (4000 IU/d) vs. placebo during resistance training	4000 IU/d	12 weeks	25(OH)D ↑, PTH ↓ in VD; peak power ↑ at 4 weeks; waist-to-hip ratio inversely correlated with 25(OH)D change	25(OH)D: increased (*P* < 0.05); PTH: decreased (*P* < 0.05); Peak power: increase at 4 weeks significant (*P* < 0.05); Correlation (R^2^ = 0.205; *P* = 0.02) between change in 25(OH)D and change in WHR	(91)
Human	40 postmenopausal women with overweight/obesity undergoing water-based aerobic training + vitamin D	Water-based aerobic training + vitamin D3 (WTD), training alone (WT), vitamin D3 alone (D), control	–	8 weeks	BMI ↓, handgrip strength ↑, gait speed ↑ in WTD; balance and gait improved in WT and WTD; vitamin D alone limited effect	BMI: WTD post-intervention ∼44.0 kg/m^2^ (CI 38.5–49.5) vs. D ∼47.7 (CI 44.0–51.4) vs. CON ∼46.4 (CI 43.0–49.8), significant reductions (*p* = 0.005–0.001)	(92)
Human	198 adults middle-aged/older with overweight/obesity and type 2 diabetes	Progressive resistance training (PRT) ± whey protein + vitamin D3 (2000 IU/day)	2000 IU/day	24 weeks	HbA1c, HOMA2-IR unchanged; IL-10 ↑, TNF-α↑, 30-s sit-to-stand ↑ in PRT + ProD	FPG improved in PRT versus PRT + ProD (net difference, 0.6 mmol/L [95% CI, 0.1, 1.0], *P* = 0.018), while interleukin IL-10 (61% [95% CI 31%, 92%], *P* < 0.001), tumor necrosis factor-α (16% [95% CI, 3%, 29%], *p* = 0.015) and 30-s sit-to-stand performance (number, 1.0 [95% CI, −0.05, 1.5], *p* = 0.047) increased in PRT + ProD versus PRT	(93)
Human	220 adults post-bariatric surgery (RYGB/SG) with vitamin D loading phase and supplementation	Vitamin D loading + ongoing VitD, calcium, protein supplementation + exercise	16,000 IU/wk and 1000 mg calciummonocitrate/ d after surgery	24 months	Bone turnover markers (CTX, P1NP) ↓; aBMD decline less pronounced; lean body mass preserved	Cross-linked C-telopeptide (CTX, 82.6% versus 158.3%), 25-OH vitamin D (13.4% versus 18.2%), phosphate (23.7% versus 32%, *p* < 0.001 for all), procollagen type 1 amino-terminal propeptide (P1NP, 12% versus 41.2%), intact parathyroid hormone (iPTH, −17.3% versus −7.6%), and Dickkopf-1 (−3.9% versus −8.9%, *p* < 0.05 for all)	(94)

↓ Reduction; ↑ Increase. BMD, bone mineral density; PTH, parathyroid hormone; ATD, aquatic training + vitamin D3; AT, aquatic training + placebo; D, vitamin D3 only; CON, control group; WHR, waist-to-hip ratio; VD, vitamin D; RT, resistance training; VRT, vitamin D + resistance training; 25(OH)D, 25-hydroxyvitamin D; PRT, progressive resistance training; ProD, protein + vitamin D supplementation; aBMD, areal bone mineral density; RYGB, Roux-en-Y Gastric Bypass; SG, sleeve gastrectomy; CTX, cross-linked C-telopeptide; P1NP, procollagen type 1 amino-terminal propeptide; IL-10, interleukin-10; TNF-α, tumor necrosis factor-alpha; BMI, body mass index; T2D, type 2 diabetes.

Another work investigated the efficacy of vitamin D supplementation in augmenting the outcomes of a 12-weeks multimodal exercise regimen among individuals with overweight or obesity suffering from vitamin D deficiency. Vitamin D significantly elevated serum 25(OH)D levels; however, it did not yield improvements in the primary endpoint of gait velocity or in most functional and metabolic metrics. Nonetheless, supplementation demonstrated a modest enhancement in stair-climbing capacity and, when coupled with exercise, led to reductions in waist circumference and waist-to-hip ratio, indicating targeted benefits for central adiposity and lower-body strength. Exercise alone resulted in anticipated advancements, whereas vitamin D supplementation in isolation exhibited negligible functional effects. The results suggest that vitamin D does not universally enhance exercise responsiveness but may exert influence over specific areas such as muscular power and abdominal fat distribution in individuals with deficient baseline vitamin D levels (89). This pilot randomized, double blind, placebo-controlled trial is methodologically well designed, particularly in its use of blinding and placebo control, which reduces performance and detection bias and strengthens internal validity. The focus on vitamin D deficient older adults increases biological relevance and the combination of supplementation with exercise is appropriate for the outcomes assessed, including physical function, body composition, and metabolic markers. Statistically, however, the pilot nature of the study implies a small sample size, limiting power and making the trial more suitable for feasibility and effect size estimation than for hypothesis testing. The analysis includes multiple outcomes, and without strong correction for multiple comparisons, the risk of chance findings is increased, especially for secondary metabolic endpoints. While randomization is a strength, limited multivariable adjustment and short intervention duration constrain the ability to fully account for confounding and to observe longer-term metabolic effects. Overall, the results are directionally consistent and useful for informing larger trials, but the reliability of the effect estimates is modest and the conclusions should be viewed as preliminary rather than definitive.

The purpose of a study was to explore the extent to which high-dose vitamin D and resistance training can alleviate obesity-associated metabolic and muscular impairments in p62-deficient murine models. Vitamin D supplementation resulted in enhanced serum 25(OH)D concentrations and mitigated adipose tissue enlargement, while resistance training and the synergistic intervention fostered improvements in glycemic regulation and diminished splenic mass, thereby indicating potential anti-inflammatory properties. Importantly, skeletal muscle functionality was compromised solely in the untreated murine subjects with obesity, suggesting that vitamin D maintained functional capacity in the absence of structural adaptations within the musculature. Resistance training was found to augment insulin sensitivity to a greater degree than vitamin D administered independently. Neither intervention produced significant alterations in muscle fiber dimensions or pivotal signaling proteins associated with hypertrophy and inflammation. In summary, vitamin D might demonstrate a decelerating effect on the progression of obesity and the preservation of muscular function, while optimal enhancements in metabolic parameters necessitated the incorporation of resistance training (90). This animal study is methodologically strong in its use of a controlled experimental design, genetically defined p62-deficient mice with obesity, and tightly regulated dosing of vitamin D and resistance exercise, which together reduce variability and support internal validity of the findings. The inclusion of appropriate control groups and objective measures of body composition and muscle function strengthens the statistical signal and makes the reported effects coherent and biologically plausible. Nevertheless, the sample size per group is relatively small, as is common in animal experiments, which can inflate effect sizes and limit the precision of estimates, particularly when multiple outcomes are analyzed. The statistical analyses rely largely on group comparisons without extensive modeling or adjustment for repeated testing, increasing the risk of false-positive results. Most importantly, the use of a specific genetic obesity model and high-dose vitamin D limits external validity, making direct translation to human obesity uncertain. Overall, the results are likely reliable within the experimental context of this mouse model, but their applicability to humans should be interpreted cautiously and viewed as mechanistic rather than clinically definitive.

Another trial investigated the extent to which vitamin D supplementation may augment physiological adaptations to resistance training in adults characterized by overweight and obesity, particularly those presenting with deficient baseline vitamin D levels. The administration of supplementation effectively elevated serum 25(OH)D concentrations and inhibited parathyroid hormone (PTH) levels; however, it yielded only marginal additional benefits when integrated with resistance training. Notably, vitamin D demonstrated a distinctive enhancement in peak power at the 4-weeks mark, implying an early ergogenic effect that may be associated with improved calcium metabolism. Furthermore, elevated vitamin D status was found to correlate with a decrease in waist-to-hip ratio, suggesting its potential significance in addressing central adiposity. Nevertheless, no substantial advancements were noted in terms of strength, glucose tolerance, or body composition beyond those accomplished exclusively through resistance training. These data underscored the notion that while vitamin D may exert an influence on certain performance metrics, it does not significantly amplify comprehensive training adaptations within this demographic (91). This study benefits from a controlled resistance training intervention and the inclusion of objective outcomes such as body composition, muscle performance, and glucose tolerance, which strengthens internal validity and aligns well with the proposed physiological mechanisms of vitamin D action. The randomized design supports causal interpretation, but from a statistical standpoint the relatively small sample size limits power, particularly for metabolic outcomes like glucose tolerance that typically show high interindividual variability. Baseline vitamin D status is considered, which is a methodological strength, yet subgroup analyses further reduce effective sample size and increase the risk of unstable estimates. The analytical approach relies mainly on between-group and pre–post comparisons with limited adjustment for potential confounders and without clear correction for multiple testing across several endpoints, raising the possibility of type I error. Adherence to supplementation and training is reported but not incorporated quantitatively into the analysis, which may attenuate or obscure true effects. Overall, the results suggesting modest benefits of vitamin D during resistance training are biologically plausible and directionally consistent, but the evidence is moderate in reliability and should be interpreted as exploratory rather than conclusive.

A study evaluated the combined and independent impacts of water-based aerobic exercise and vitamin D3 supplementation on physical fitness in postmenopausal women with overweight and obesity. The combination of both interventions resulted in the most significant advancements in body mass index (BMI), handgrip strength, balance, and gait velocity, thereby illustrating a pronounced synergistic effect on neuromuscular capabilities. Water-based exercise conducted in isolation also resulted in substantial improvements in strength and balance, whereas the administration of vitamin D alone produced more modest, selective enhancements in handgrip strength without significantly improving other functional measures. The outcomes underscore the efficacy of aquatic exercise as a viable low-impact approach for augmenting physical fitness within this demographic, while also indicating that the concurrent administration of vitamin D may enhance these physiological adaptations. Nevertheless, vitamin D administered in isolation is inadequate for achieving comprehensive functional enhancement (92). This randomized controlled trial has several strengths, most notably its experimental design, which supports causal inference, and its focus on a well-defined population with overweight and obesity, reducing clinical heterogeneity. The combination of vitamin D3 supplementation with a standardized aerobic water-based training program is biologically plausible, and the use of objective physical fitness indices strengthens outcome validity. From a statistical perspective, however, the sample size appears modest, which may limit power and increase uncertainty around effect estimates, especially if multiple fitness outcomes were tested without adequate correction for multiple comparisons. Details on randomization procedures and allocation concealment are limited, making it harder to fully assess risk of selection bias, and although adherence to training and supplementation is reported, residual variability in compliance may still influence results. The analyses largely rely on pre–post and between-group comparisons with limited multivariable adjustment, so potential confounders such as baseline fitness level, sun exposure, or dietary intake may not be fully controlled. Overall, the findings are consistent with physiological expectations and likely reliable in terms of direction of effect, but the magnitude of benefit should be interpreted cautiously, as the statistical evidence is supportive rather than definitive.

A 24-weeks randomized experiment investigated whether the addition of whey protein and vitamin D could amplify the advantages of progressive resistance training among individuals with overweight or obesity and type 2 diabetes. The combined intervention failed to boost enhancements in glycemic control, insulin sensitivity, or body composition beyond the gains attained through resistance training alone. While the supplementation did elevate anti-inflammatory markers like IL-10 and slightly improved sit-to-stand capabilities, these benefits did not lead to noteworthy metabolic or functional superiority. Resistance training continued to be the key catalyst for advancements in HbA1c and cardiometabolic well-being. In summary, the combination of protein and vitamin D supplementation did not significantly elevate the physiological response to resistance training, indicating a limited additive benefit for most clinical outcomes within this demographic (93). This 24-weeks randomized controlled trial has strong methodological credibility, particularly due to its randomized design, relatively long intervention period, and the combination of resistance training with supplementation, which aligns well with the physiological outcomes assessed. The use of multiple clinically relevant endpoints, including HbA1c, body composition, muscle strength, and cardiometabolic markers, adds breadth, and the progressive resistance training protocol is well standardized, reducing performance bias. Statistically, however, the study may be underpowered for some secondary outcomes, as the sample size appears optimized for glycemic control rather than the full range of cardiometabolic endpoints, increasing the risk of null findings due to limited power. While between-group analyses are appropriate, limited adjustment for baseline differences and multiple comparisons raises concern about inflated type I error, particularly given the number of outcomes examined. Adherence to both supplementation and exercise is reasonably reported but still subject to variability, which may dilute true effects. Overall, the primary findings on glycemic control and muscle function are reasonably reliable, but conclusions regarding broader cardiometabolic risk reduction should be interpreted cautiously and viewed as suggestive rather than definitive.

An imaginative 24-months interventional exploration scrutinized whether a vitamin D loading phase, succeeded by postoperative supplementation of vitamin D, calcium, and protein alongside regimented physical activity, curtails bone depletion post-bariatric surgery. In contrast to the non-intervention cohort, those receiving supplements revealed considerably lesser spikes in bone turnover indicators, such as CTX and P1NP, while showing diminished declines in lumbar spine, hip, and overall body aBMD. The downturns in lean body mass and trabecular bone score were notably moderated. Enhancements in biochemical indicators like lowered PTH and improved sclerostin levels further illuminated superior skeletal governance. These revelations illustrated that a harmonious blend of nutritional reinforcement and exercise may proficiently decelerate postoperative bone decline and safeguards musculoskeletal vitality, emphasizing the critical nature of multifaceted approaches in the realm of bariatric aftercare (94). The BABS study benefits from a prospective interventional design and the use of validated bone markers and BMD outcomes, which strengthens the biological and clinical relevance of its findings after bariatric surgery. However, the relatively small sample size limits statistical power, especially for detecting moderate effects and controlling for key confounders such as baseline vitamin D status, sex, and type of surgery. The analyses rely mainly on repeated group comparisons without extensive multivariable adjustment or correction for multiple testing, increasing the risk of chance findings. Adherence to supplementation and exercise is imperfectly measured, and loss to follow-up may introduce bias, further weakening precision. Overall, the results are plausible and directionally consistent with current knowledge, but they should be interpreted cautiously, as the evidence is supportive rather than robustly reliable for definitive clinical guidance.

Based on the collective evidence from these studies, vitamin D supplementation combined with exercise may appear beneficial for improving musculoskeletal health, body composition, and certain metabolic parameters in both humans and preclinical models, but the reliability of these findings varies. Across the human trials, consistent increases in serum 25(OH)D were observed with doses of 2000–4000 IU/day, and higher weekly loading doses post-bariatric surgery (16,000 IU/week) effectively mitigated bone loss. Exercise type matters: resistance training, progressive resistance training, and water-based aerobic or aquatic training enhanced muscle function, strength, and balance, while multi-modal exercise contributed to reductions in waist circumference and improved stair climb performance when combined with vitamin D. In animal models, high-dose vitamin D with resistance training preserved skeletal muscle and improved glucose metabolism, supporting mechanistic plausibility. However, limitations are notable. Sample sizes were often small, intervention durations relatively short (8–24 weeks in most human studies), and adherence to supplementation and exercise protocols variably reported, which reduces statistical power and precision. Many studies relied on pre–post comparisons with limited multivariable adjustment and minimal correction for multiple outcomes, increasing the risk of false-positive findings. Additionally, heterogeneity in populations, from postmenopausal women to older adults with obesity and type 2 diabetes, and genetically modified mice, limits direct generalizability. Overall, the evidence suggests that daily vitamin D supplementation of 2000–4000 IU combined with resistance or water-based aerobic training is likely effective for improving musculoskeletal and some metabolic outcomes, but the quantitative magnitude of benefit remains uncertain, and larger, longer-term, well-powered trials with rigorous adherence monitoring are needed to confirm these effects.

A comparison of intervention duration among studies summarized in Section “4.4 Evidence suggesting a potential additive interaction on muscle metabolism and function” and Table 2 highlights important temporal differences in the effects of combined vitamin D supplementation and exercise. Short-term interventions, most commonly ranging from 8 to 16 weeks, consistently demonstrate rapid improvements in metabolic markers, body composition, and inflammatory profiles, with exercise serving as the principal driver of change. Within these shorter trials, vitamin D supplementation frequently may enhance biochemical sufficiency and may contribute selectively to outcomes such as reductions in central adiposity, modest improvements in muscle performance, or regulation of endocrine markers; however, its additive effect beyond exercise alone remains variable and often limited to specific subgroups, particularly individuals with baseline deficiency or higher adiposity. In contrast, longer-duration studies and extended experimental models suggest that sustained combined exposure may influence deeper physiological processes, including mitochondrial adaptations, inflammatory signaling pathways, and neurovascular–muscle axis integration, although consistent clinical superiority over exercise alone has not been universally demonstrated. The apparent discrepancy between short-term metabolic responsiveness and less consistent long-term clinical outcomes likely reflects differences in mechanistic timelines, where early improvements arise from acute exercise-induced metabolic shifts, whereas vitamin D–related effects may require prolonged normalization of endocrine and inflammatory environments. Additionally, longer trials often involve more heterogeneous populations and greater variability in adherence, which may attenuate observable benefits. Taken together, the evidence indicates that intervention duration acts as a key moderator of outcomes, with short-term studies capturing rapid functional and metabolic adaptations and longer-term studies necessary to evaluate structural, neurocognitive, or systemic endpoints; however, definitive synergistic effects remain conditional on baseline vitamin D status, exercise intensity, and participant phenotype.

Applying the predefined classification framework, exercise-induced improvements in muscle strength, mitochondrial function, glucose utilization, and insulin responsiveness in individuals with overweight and obesity are empirically supported by human interventional studies. Functional outcomes such as increased muscle strength, improved physical performance, and enhanced glucose tolerance following structured training are consistently demonstrated in obesity cohorts.

Vitamin D–related influences on calcium handling, mitochondrial signaling (AMPK–PGC-1α), mTOR activation, and myostatin inhibition are supported in human deficiency states and in non-obesity populations but remain insufficiently confirmed within obesity-specific exercise trials. These interactions are therefore categorized as supported in related human contexts or mechanistically inferred, depending on the outcome domain. Evidence for a true additive or synergistic enhancement of exercise-induced muscle remodeling through vitamin D supplementation in obesity-specific populations remains limited and heterogeneous. Accordingly, the proposed amplification of exercise responsiveness by vitamin D in skeletal muscle should be interpreted as a biologically plausible but not yet empirically validated component of the neurovascular–muscle axis in obesity.

Similarly, the 2025 literature advances the conceptual precision of the neurovascular–muscle axis by clarifying that mechanistic overlap does not uniformly translate into additive clinical magnitude. Instead, synergy appears domain-specific: strongest in antioxidant and neurotrophic signaling (preclinical evidence), moderate in muscle function under deficiency conditions, and least consistent in anthropometric and glycemic endpoints. This graded responsiveness supports a hierarchical interpretation of vitamin D–exercise interaction, wherein exercise remains the primary metabolic driver and vitamin D acts as a context-sensitive amplifier whose efficacy depends on baseline deficiency, tissue-specific responsiveness, and intervention duration (15–17, 49).

Importantly, the neurovascular–muscle axis should be interpreted as a graded evidence model rather than a uniformly validated systems map. While exercise-driven improvements in metabolic and vascular domains are supported by human data in obesity, vitamin D–exercise interactions across neural, vascular, and muscular systems remain partially mechanistic and population-dependent. The visual distinction between validated and inferred pathways in Figure 1 is therefore intentionally reflected in the narrative classification throughout Section “2 Molecular and physiological basis of the vitamin D–exercise interaction” – Section “4 Effects on muscle metabolism and functional adaptation.”

To summarize:

1. Exercise is the principal driver of improvements in muscle strength, mitochondrial function, and metabolic flexibility.
2. Vitamin D plays a recognized role in calcium handling and muscle physiology, but its capacity to augment training adaptations appears modest.
3. Some studies suggest selective benefits (e.g., peak power, central adiposity, endocrine markers), particularly in deficient individuals.
4. Evidence for sustained or clinically meaningful synergistic effects remains insufficient.

## 5 Vitamin D with exercise and neurovascular health

### 5.1 Neurocognitive and neurovascular health

**FIGURE 3:**
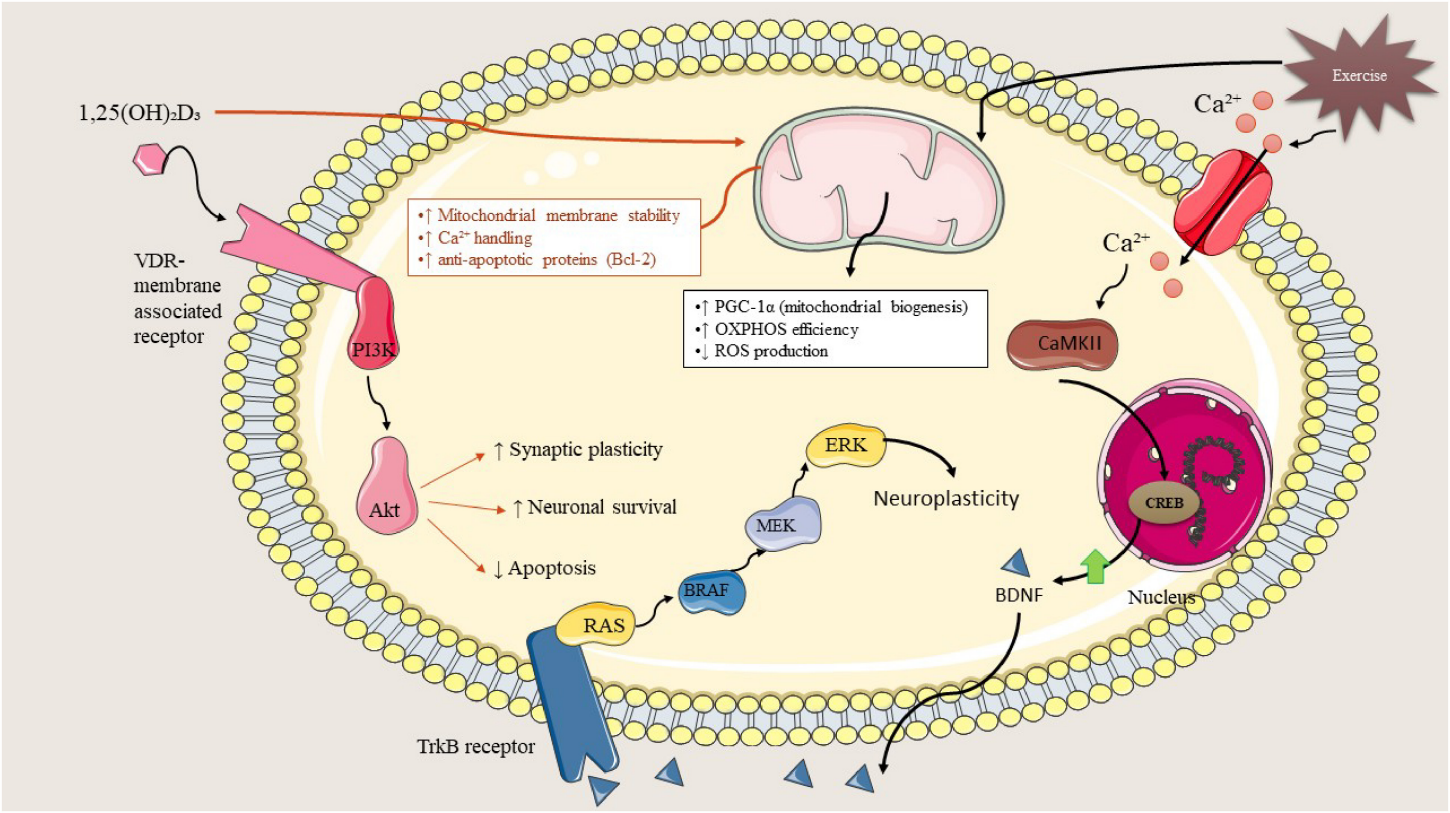
Convergent intracellular pathways through which vitamin D and exercise promote neuronal plasticity and survival. 1,25(OH)2D3 activates membrane-associated VDR, stimulating PI3K–Akt signaling to enhance synaptic plasticity, neuronal survival, and anti-apoptotic processes. Exercise-induced Ca^2+^ influx activates CaMKII and CREB-dependent transcription of neurotrophic genes. Both vitamin D and exercise improve mitochondrial stability, Ca^2+^ handling, and anti-apoptotic signaling, while exercise further increases PGC-1α–driven mitochondrial biogenesis, OXPHOS efficiency, and reduces ROS. BDNF activation of TrkB initiates the RAS–BRAF–MEK–ERK cascade, supporting neuroplasticity. Together, these pathways converge to enhance neuronal function and resilience.

The interplay between vitamin D and physical exercise suggests substantial potential for the enhancement of neurocognitive and neurovascular health, particularly within populations with overweight or obesity (Figure 3). The underlying mechanisms encompass the restoration of neuronal density, the preservation of synaptic integrity, the attenuation of neuroinflammation, and the improvement of vascular function.

To maintain consistency with the evidence-classification framework introduced in Section “2 Molecular and physiological basis of the vitamin D–exercise interaction,” mechanistic interactions discussed in this section are explicitly identified as (i) empirically supported in humans with obesity, (ii) supported in related human contexts, or (iii) mechanistically inferred from animal, cellular, or indirect physiological evidence.

### 5.2 Combination of vitamin D and exercise in neuronal density and synaptic integrity

Preclinical investigations suggest that the integration of physical exercise with vitamin D supplementation can facilitate the restoration of neuronal architecture in critical regions of the brain. Physical exercise (PE) exerts multifaceted neuroprotective effects and is increasingly recognized for its potential to counteract neurodegenerative disorders through multiple interconnected mechanisms (95). PE is defined as structured, repeated aerobic or anaerobic activity with specified frequency, duration, and intensity, designed to improve or maintain physical fitness. Regular engagement in PE has been shown to slow age-related cognitive decline, enhance cerebral blood flow, and support neuronal plasticity. Neuroimaging and clinical studies demonstrate that PE increases gray matter volume, particularly in the frontal cortex and hippocampus, and elevates levels of neurotrophic factors such as brain-derived neurotrophic factor (BDNF) (96). Beyond neurotrophic support, adapted PE functions as a potent anti-inflammatory intervention. It reduces systemic and CNS-resident proinflammatory cytokines, a process mediated both by direct effects on immune cells (“exercise immunology”) and by myokines, bioactive molecules released by contracting skeletal muscle. Over 600 myokines have been identified, many of which remain to be fully characterized. Certain myokines, including BDNF, IL-6, irisin, cathepsin B, and IGF-1, can cross the blood–brain barrier and mediate muscle–brain cross-talk, exerting neurotrophic, anti-inflammatory, and regulatory effects on the CNS (95). Recent evidence suggests a complementary effect between PE and vitamin D in neuroprotection (97). Vitamin D supports the production and action of several myokines and neurotrophic factors, enhancing their effects on neuronal health. For instance, vitamin D modulates BDNF expression, mitigates molecular derangements in neurodegeneration, and promotes neuroprotective outcomes in vitamin D-deficient conditions. Elevated BDNF levels facilitate the shift of microglia from the proinflammatory M1 phenotype to the anti-inflammatory M2 phenotype, reducing the secretion of cytokines such as IL-1β and TNFα. Vitamin D also may enhance the expression of irisin, IL-6, cathepsin B, and IGF-1, while regular PE increases vitamin D receptor (VDR) expression in key cognitive and emotional brain regions, including the hippocampus and prefrontal cortex. This creates a positive feedback loop, enhancing neuronal responsiveness to vitamin D and potentiating the neuroprotective benefits of exercise (49). At the molecular level, both PE and vitamin D converge on shared anti-inflammatory and antioxidant pathways. They inhibit NF-κB signaling (98), a central regulator of proinflammatory gene expression, and activate the Nrf2 pathway, which upregulates antioxidant enzymes such as superoxide dismutase, glutathione peroxidase, and catalase (99). Vitamin D additionally may enhance heme oxygenase-1 and GPX4 expression and reduces ferroptosis in aging brains (99), collectively mitigating oxidative stress and neuroinflammation. Experimental models indicate that combining PE with vitamin D produces might have some effects on cognitive function, blood–brain barrier integrity, and mitochondrial efficiency. Overall, these overlapping molecular targets support the hypothesis that PE and vitamin D act together to preserve neuronal function, reduce inflammation and oxidative stress, and potentially prevent or slow the progression of neurodegenerative diseases (49).

### 5.3 Reduction of neuroinflammation and improvement of mood and cognitive performance

The role of neuroinflammation in neurodegenerative diseases remains a central question, with debate over whether it is a primary cause or a consequence of neuronal damage (100). In the central nervous system (CNS), inflammation is initially a protective physiological response, mediated primarily by resident immune cells, microglia, which detect harmful stimuli such as infections or trauma and help maintain cellular homeostasis. Upon activation, microglia trigger a neuroinflammatory response characterized by the release of reactive oxygen species (ROS), reactive nitrogen species, cytokines, and chemokines, alongside dysfunction of the blood–brain barrier (BBB). While normally transient, an inappropriate or prolonged response leads to chronic neuroinflammation, creating a self-reinforcing cycle that can precipitate neurodegenerative processes (100). This chronic inflammatory state is further amplified by interactions between CNS-resident cells, including microglia, astrocytes, oligodendrocytes, pericytes, and endothelial cells, and peripheral immune cells infiltrating the CNS. Peripheral immune infiltration can be triggered by systemic inflammation, such as that induced by pathogens or endotoxins, which activates microglia to release inflammatory mediators and recruit additional immune cells (101). This interaction between systemic and local compartments establishes a vicious, mutually reinforcing loop, often referred to as a systemic–local detrimental loop. Within this environment, aberrant signaling cascades involving pattern recognition receptors (PRRs), toll-like receptors (TLRs), the NLRP3 inflammasome, receptors for advanced glycation end products (RAGE), and damage-associated molecular patterns (DAMPs) amplify inflammation, while persistent ROS, cytokines, chemokines, protein aggregates, ATP, and mitochondrial DNA from damaged neurons sustain immune activation (101).

A key consequence of chronic neuroinflammation is the massive release of proinflammatory cytokines, including TNFα, IL-6, IL-1β, IL-23, IFNγ, and GM-CSF. These molecules exacerbate tissue damage through multiple mechanisms, such as promoting pathogenic T cell activity, facilitating macrophage infiltration, and impairing protein clearance (102–104). The resulting pathological environment drives synaptic dysfunction, neuronal loss, and protein aggregation, exemplified by amyloid-beta accumulation in Alzheimer’s disease (AD) and α-synuclein aggregation in Parkinson’s disease (PD). In AD, chronically activated microglia fail to clear amyloid-beta efficiently, reinforcing plaque formation and neuronal damage. In PD, α-synuclein oligomers further activate microglia, while excess ROS, nitric oxide, and TNFα mediate neurotoxicity. Peripheral immune cells, particularly CD4+ and CD8+ T cells, infiltrate the CNS, sometimes preceding pathological protein deposition, highlighting the interplay between systemic and local immune responses (102–104). Additional factors, such as gut–brain axis dysregulation and gut microbiota alterations, further exacerbate neuroinflammation by increasing BBB permeability and facilitating immune cell infiltration. Overall, these processes form a self-amplifying, mutual-promotion loop in which systemic and CNS inflammation reinforce each other, ultimately contributing to the progression of diverse neurodegenerative diseases. Despite differences in disease-specific mechanisms, protein aggregates, and genetic backgrounds, chronic neuroinflammation emerges as a shared pathological hallmark linking neuronal injury, immune dysregulation, and disease exacerbation (102–104). Combined administration of vitamin D and physical exercise might cause a reduction in neuroinflammatory markers. In a murine model, the cohort receiving both exercise and vitamin D displayed elevated levels of IL-10, diminished hippocampal TNF-α, and a reduction in astrocyte activation (105). In human subjects, the combination of supplemental vitamin D and aerobic exercise resulted in increased BDNF, a decrease in pro-inflammatory cytokines (IL-6, TNF-α), and an enhancement of neurotrophin profiles that surpassed the effects of either intervention in isolation (106). These anti-inflammatory and neurotrophic outcomes may play a significant role in the enhancement of mood and cognitive functioning (15, 106).

### 5.4 Possible mediation via BDNF, NO signaling, and VDR expression

The facilitation of BDNF signaling is deemed pivotal to the neuroprotective benefits conferred by vitamin D and physical exercise (15). Vitamin D possesses the capacity to influence oxidative stress as well as vascular functionality, possibly implicating NO signaling pathways. In a rat model representative of Parkinson’s disease, the combination of vitamin D and exercise resulted in a decrease in cerebral oxidative stress while simultaneously enhancing the expression of VDR (107). VDR is localized within both neuronal and glial populations, particularly in regions such as the hippocampus and cortex, thereby facilitating the direct actions of vitamin D within the central nervous system (15, 108). Physical exercise may promote an increase in VDR expression within the brain, thereby amplifying the neuroprotective signaling mediated by vitamin D (15). A research scrutinized the synergistic impacts of vitamin D supplementation and aerobic physical activity on the hypothalamic arcuate (ARC) and ventromedial (VMH) nuclei in rats with obesity via monosodium glutamate (MSG) administration. A total of five distinct groups were subjected to evaluation: control, MSG-induced obesity, vitamin D-treated, exercise-trained, and a combination of vitamin D and exercise. The administration of MSG resulted in both neuronal and glial cell degeneration within the ARC, along with a decrement in neuronal populations within the VMH. Exercise interventions led to a reduction in visceral adiposity and an enhancement in glial cell proliferation, while the integrative approach of vitamin D and exercise not only fully reinstated neuronal and glial cell densities in the ARC but also augmented neuronal counts within the VMH. The data demonstrated that early, integrative therapeutic strategies may effectively ameliorate hypothalamic damage induced by obesity and potentially enhance the regulation of satiety (109). Another investigation assessed the impact of vitamin D supplementation (2,000 IU/day for a duration of 12 weeks) in conjunction with either indoor (treadmill) or outdoor (jogging) physical activity on depressive disorders in a cohort of 75 Iranian women with obesity. Depressive symptoms were evaluated utilizing the Beck Depression Inventory as a measurement tool. The findings indicated that outdoor exercise accompanied by vitamin D supplementation resulted in a significant reduction of severe depression scores by 50%, decreasing from 33.26 ± 2.12 to 16.73 ± 1.38. Moreover, indoor exercise coupled with vitamin D supplementation, as well as exercise in isolation, also contributed to the amelioration of depressive symptoms, transitioning from severe to moderate levels. The study underscored the notion that both vitamin D and physical activity, particularly in outdoor settings, work synergistically to mitigate depression in women with obesity and address the issue of vitamin D deficiency (110). The aim of a study was to explore a 12-weeks regimen of BMI-adjusted vitamin D3 supplementation among individuals diagnosed with Parkinson’s disease who were undergoing deep brain stimulation, focusing on the evaluation of physical performance, inflammatory markers, and vitamin D metabolites. A total of thirteen subjects were administered vitamin D3, whereas sixteen individuals were assigned to receive a placebo. The administration of vitamin D3 resulted in an elevation of serum 25(OH)D3 to optimal concentrations (>30 ng/mL) and led to a significant augmentation in vitamin D metabolites. Improvements in functional performance were observed, as demonstrated by enhanced scores in the Timed Up and Go and 6-Minute Walk Test assessments. Notable trends indicative of reduced inflammation was documented. The results imply that attaining optimal vitamin D levels may enhance physical functionality and potentially diminish the risk of falls among patients with Parkinson’s disease, thereby endorsing the implementation of targeted supplementation based on body mass index (BMI) (111). The synergistic interaction between vitamin D and physical exercise plays a significant role in enhancing neurocognitive and neurovascular health through a multitude of mechanisms: augmenting neurotrophic factors, attenuating neuroinflammation, modulating redox equilibrium, and promoting VDR-mediated signaling pathways. These beneficial effects may contribute to the restoration of neuronal density, the maintenance of synaptic integrity, the enhancement of mood and cognitive functions, and the support of vascular integrity. Despite the robust preclinical evidence, it is imperative to conduct targeted clinical trials within overweight and obesity cohorts to assess the long-term safety of these interventions. While preclinical and clinical studies suggest convergent neuroprotective effects of vitamin D and exercise, much of the causal evidence derives from populations without obesity or disease-specific, underscoring the need for obesity-focused trials to confirm translational relevance.

Within the neurovascular domain, exercise-induced improvements in endothelial function, cerebral perfusion, and cardiometabolic risk markers in obesity are empirically supported in human studies. Exercise-mediated enhancement of vascular reactivity and reductions in systemic inflammation are well-established in overweight and obese cohorts. Vitamin D–related modulation of endothelial nitric oxide production, blood–brain barrier integrity, and neurotrophic signaling (e.g., BDNF regulation) is supported by preclinical studies and selected human observational data but lacks consistent confirmation in obesity-specific randomized exercise trials. These interactions are therefore best classified as mechanistically inferred or supported in related human contexts. The integrated neurovascular–muscle axis model proposed herein should therefore be interpreted as a conceptual framework that integrates empirically supported exercise effects with biologically plausible vitamin D–mediated modulation, rather than as a fully validated systems-level interaction in obesity.

Taken together:

1. Exercise exerts well-established neuroprotective and neurovascular benefits, including enhanced BDNF signaling and improved cerebral perfusion.
2. Vitamin D influences neuroimmune and endothelial pathways and may interact with exercise-related signaling mechanisms.
3. Most evidence for synergistic neurocognitive effects derives from experimental models.
4. Clinical confirmation of additive benefit in obesity-specific populations is currently lacking.

## 6 Cardiometabolic and hepatic axis

The interaction between vitamin D and physical exercise exerts profound influences on cardiometabolic and hepatic health, especially within subjects with overweight and obesity. Research indicates that this synergistic relationship can positively affect lipid profiles, hepatic enzyme activity, and vascular risk determinants (71). Furthermore, vitamin D and physical exercise modulate hormones related to appetite and leptin sensitivity, thereby contributing to the maintenance of energy homeostasis and the regulation of body weight (70). Empirical studies demonstrate that vitamin D amplifies leptin receptor signaling in adipose tissue, while both aerobic and resistance training enhance leptin responsiveness, consequently mitigating hyperphagia and metabolic dysregulation (70, 112). Furthermore, the biomarkers associated with non-alcoholic fatty liver disease (NAFLD), such as hepatic steatosis, liver lipid content, and inflammatory responses, appear to exhibit responsiveness to interventions involving vitamin D and exercise. Experimental studies utilizing animal models indicate that the combination of vitamin D supplementation and aerobic exercise leads to a reduction in hepatic triglyceride accumulation, an enhancement of insulin sensitivity, and a mitigation of inflammatory signaling within the hepatic tissue (78, 113). These outcomes underscore the interconnectedness of liver, muscle, and adipose tissue signaling pathways, wherein vitamin D and physical exercise function synergistically to improve glucose and lipid metabolism, diminish adipose tissue inflammation, and re-establish hepatic homeostasis (78).

A study examined the synergistic effects of vitamin D supplementation in conjunction with aerobic interval training on women with obesity experiencing perceived myalgia. A total of forty-five women diagnosed with vitamin D deficiency were allocated into three distinct groups: those receiving aerobic exercise alongside vitamin D supplementation, those receiving vitamin D exclusively, and those engaging in exercise alone, all while adhering to calorie-restricted diets. The primary outcomes assessed included pain intensity measured via the Visual Analog Scale (VAS), serum levels of vitamin D, functional capacity evaluated through the Cooper 12-Minute Walk Test, and overall quality of life as determined by the SF-36 instrument. The combined intervention yielded statistically significant improvements in pain alleviation, functional capacity, serum vitamin D levels, and quality of life as compared to either intervention administered in isolation. The exercise-only group exhibited enhancements in functional capacity; however, it demonstrated a comparatively minimal effect on pain relief and serum vitamin D concentrations. The findings exhibited that the integrative approach of vitamin D supplementation combined with aerobic exercise is more efficacious in addressing obesity-related myalgia and in promoting both physical and mental well-being (114). A 14-weeks investigation assessed the impact of calcium and vitamin D supplementation in conjunction with energy-restricted dietary regimens and resistance training on post-menopausal women (*n* = 128). Participants adhered to normal, low-calorie, higher-carbohydrate, or low-calorie, higher-protein dietary protocols while receiving either a placebo, calcium, or calcium combined with vitamin D. The exercise regimen may result in enhancements in strength, aerobic capacity, and blood lipid profiles across all groups. The incorporation of calcium into a high-carbohydrate diet facilitated a more significant reduction in body mass, whereas the combination of calcium and vitamin D contributed to the preservation of fat-free mass and a decrease in fat percentage. No statistically significant differences were detected when calcium or calcium plus vitamin D were incorporated into the high-protein diet. The data proved that particular combinations of supplementation and dietary strategies can optimize outcomes related to body composition during periods of energy restriction and exercise interventions (115). Another work assessed the impact of a 6-weeks regimen of vitamin D supplementation (2,000 IU/day) on hepatic enzyme levels and lipid profiles among a cohort of 22 overweight females diagnosed with non-alcoholic fatty liver disease (NAFLD) who engaged in eccentric exhaustive exercise (EEE). Administration of vitamin D substantially diminished body weight, body mass index (BMI), body fat percentage, and the waist-to-hip ratio. Subsequent to EEE, there was an elevation in liver enzymes [alanine aminotransferase (ALT), aspartate aminotransferase (AST), gamma-glutamyl transferase (GGT)] and lipid markers [total cholesterol (TC), triglycerides (TG), low-density lipoprotein (LDL)], whereas high-density lipoprotein (HDL) exhibited a decline. The provision of vitamin D supplementation mitigated these EEE-induced alterations, resulting in reduced hepatic enzyme levels and enhanced lipid profiles in comparison to the placebo group. These results indicated that vitamin D serves as a functional supplement capable of alleviating exercise-induced hepatic stress and dyslipidemia in overweight women with NAFLD, thereby supporting metabolic and hepatic health during periods of rigorous physical activity (116). The purpose of investigation was to assess the impact of an 8-weeks regimen of high-intensity interval training (HIIT), both with and without the adjunct of vitamin D supplementation (2,000 IU/day), on appetite-regulating hormones and body composition metrics in a cohort of 48 sedentary overweight males. The participants were stratified into groups receiving HIIT in conjunction with vitamin D, HIIT paired with a placebo, vitamin D alone, or a control condition. The combination of HIIT and vitamin D resulted in a statistically significant reduction in weight, body mass index (BMI), adiposity, and insulin concentrations, alongside an elevation in peptide YY (PYY), when compared to the vitamin D monotherapy or control groups. The HIIT intervention alone also yielded improvements in these physiological parameters; however, the increase in PYY was more pronounced in the HIIT combined with vitamin D cohort. These findings demonstrated that the synergistic effect of HIIT and vitamin D supplementation may enhance mechanisms of appetite regulation and body composition, thereby potentially contributing to improved energy homeostasis and metabolic health in sedentary overweight individuals (117). In summary, the synergistic effects of vitamin D and exercise constitute a comprehensive multi-organ therapeutic approach that optimizes cardiometabolic and hepatic health outcomes through intricate hormonal, metabolic, and inflammatory interactions.

Obesity management is inherently multifactorial and typically relies on dietary interventions, behavioral modification, pharmacotherapy, and, in selected cases, bariatric surgery. In this context, the vitamin D–exercise axis should not be viewed as a replacement for these strategies, but rather as a complementary approach that may enhance metabolic resilience, musculoskeletal integrity, and neurovascular health, particularly in individuals with limited tolerance to aggressive weight-loss interventions. Unlike pharmacological or surgical therapies, this combined strategy primarily targets functional and systemic adaptations, which may support long-term adherence and mitigate obesity-related complications independent of substantial weight loss. To synthesize the principal mechanistic and translational insights discussed above, the key conclusions are summarized below:

Exercise is the primary driver of improvements in hepatic lipid handling, insulin sensitivity, and systemic cardiometabolic regulation.
Vitamin D appears to support hepatic and vascular homeostasis through context-dependent effects on inflammation, lipid metabolism, and cellular stress responses.
Combined interventions are associated with complementary or additive improvements in hepatic steatosis, glycemic control, and inflammatory balance, particularly in metabolically compromised states.
Evidence for direct mechanistic synergy in humans remains limited, with most pathway-level insights derived from preclinical models.
Variability in baseline metabolic status, vitamin D sufficiency, and intervention design contributes to heterogeneous hepatic and cardiometabolic outcomes across studies.
Current evidence supports a targeted adjunctive role for vitamin D within exercise-centered strategies for cardiometabolic risk reduction.

## 7 Clinical and translational evidence

### 7.1 Summary of human clinical trials assessing vitamin D–exercise co-treatment

Empirical evidence from human clinical studies increasingly elucidates that the concomitant implementation of vitamin D supplementation alongside structured physical activity yields synergistic advantages across various domains, including metabolic, muscular, vascular, and neurocognitive facets in populations characterized by overweight and obesity. Numerous randomized controlled trials (RCTs) indicate that the integration of vitamin D with aerobic exercise, resistance training, or a combination thereof significantly may enhance insulin sensitivity, mitigates systemic inflammation, and promotes superior improvements in body composition compared to either intervention independently (74, 89). A randomized clinical trial investigated the efficacy of Pilates exercise, vitamin D supplementation, or a combination of both in ameliorating renal function parameters among overweight males aged 45–55 years. Over a duration of 8 weeks, participants were allocated to one of four groups: Pilates training, vitamin D supplementation, combined treatment, or a control group. The Pilates regimen was conducted thrice weekly at a moderate intensity, whereas the supplementation cohorts received a dosage of 50,000 IU of vitamin D on a weekly basis. The interventions yielded statistically significant improvements in glomerular filtration rate and reductions in levels of urea, uric acid, and creatinine, thereby indicating enhanced renal function. Importantly, the combined treatment manifested the most pronounced benefits, suggesting the existence of synergistic effects attributable to the integration of physical training and vitamin D. These data underscored the potential of non-pharmacological, lifestyle-oriented interventions for promoting renal health in overweight individuals exhibiting suboptimal vitamin D status (118). The purpose of another study was to examine the impact of Pilates exercise, vitamin D supplementation, and their synergistic effects on inflammatory biomarkers in a cohort of overweight males. Fifty-two subjects were randomly assigned to either Pilates, vitamin D, combined treatment, or control groups, and participated in 8 weeks of supervised training and/or a weekly dosage of 50,000 IU of vitamin D. The results demonstrated statistically significant elevations in the anti-inflammatory cytokine IL-10 levels and reductions in the pro-inflammatory cytokine TNF-α across all intervention cohorts. The combined treatment modality resulted in the most pronounced overall enhancements, suggesting additive anti-inflammatory properties. Notably, Pilates alone yielded a greater reduction in TNF-α compared to vitamin D alone, underscoring the efficacy of physical exercise in the modulation of systemic inflammation. Collectively, these findings imply that the integration of Pilates training with vitamin D supplementation confers superior immunomodulatory advantages in overweight men characterized by low vitamin D levels (119). A quasi-experimental investigation assessed the effects of Pilates exercise, vitamin D supplementation, and their concurrent application on vitamin D status and insulin resistance in overweight males aged 45–55 years. Participants engaged in 8 weeks of Pilates training or were administered a weekly dose of 50,000 IU of vitamin D, whereas a group receiving both interventions was also constituted. Initial evaluations revealed a significant prevalence of vitamin D deficiency among the subjects. All interventions were found to markedly elevate serum 25(OH)D levels and diminish fasting glucose, insulin, and insulin resistance. Vitamin D supplementation and the combined intervention resulted in more pronounced increases in 25(OH)D levels compared to exercise alone, while the concurrent treatment yielded the most substantial reductions in insulin levels and insulin resistance. These data indicated that the restoration of vitamin D levels, when combined with structured exercise, may have a synergistic effect on enhancing metabolic health in overweight adults (120). Collectively, these studies show that Pilates training and vitamin D supplementation, independently and especially in combination, significantly improve renal function, reduce inflammation, enhance vitamin D status, and decrease insulin resistance in overweight men. Their synergistic effects highlight the value of integrating structured exercise with vitamin D optimization to support metabolic and overall health. To facilitate interpretation of the heterogeneous clinical findings, the principal conclusions of the reviewed trials are summarized below:

Exercise consistently improves adiposity, metabolic regulation, and inflammatory status across clinical populations with overweight or obesity.
Vitamin D supplementation provides adjunctive, context-dependent benefits, most evident in individuals with deficiency, metabolic dysfunction, or higher cardiometabolic risk.
Combined interventions typically produce additive or permissive effects, while clear synergistic clinical benefit remains inconsistent across trials.
Exercise modality influences outcomes: aerobic and multimodal programs show the most consistent cardiometabolic improvements, whereas resistance training primarily supports functional and inflammatory adaptations.
Heterogeneity in baseline vitamin D status, metabolic phenotype, intervention duration, and exercise prescription contributes to variability across studies.
Current clinical evidence supports targeted co-treatment strategies rather than routine combined supplementation for all patients with obesity.

### 7.2 Effects on inflammatory cytokines, vitamin D status, renal function, and metabolic biomarkers

Co-administration of vitamin D in conjunction with physical exercise consistently results in a significant reduction of pro-inflammatory cytokines, including TNF-α, IL-6, IL-1β, and CRP in individuals with overweight or obesity. A multitude of clinical trials document enhanced decreases in CRP and IL-6 concentrations within vitamin D-exercise cohorts when compared to groups engaging solely in exercise, a phenomenon attributed to the optimization of adipose–muscle immunometabolic communication (67, 121). Furthermore, an upregulation of anti-inflammatory markers such as IL-10 has been documented in this context. Solely engaging in exercise does not consistently elevate 25(OH)D levels; however, the administration of vitamin D during training expediently restores vitamin D status and facilitates musculoskeletal adaptations through the enhancement of calcium homeostasis, mitochondrial biogenesis, and muscle fiber contractility (122). Individuals suffering from obesity demonstrate diminished bioavailability of vitamin D due to its sequestration within adipose tissue; hence, supplementation is particularly vital for this demographic. Clinical investigations assessing renal biomarkers indicate either neutral or marginally advantageous effects stemming from vitamin D-exercise interventions.

### 7.3 Limitations: dosage variability, exercise modality, population heterogeneity, and duration of interventions

Although contemporary empirical evidence underscores the therapeutic efficacy of integrating vitamin D supplementation with structured physical activity in populations characterized by overweight and obesity, numerous significant limitations attenuate the robustness and applicability of the prevailing findings. A principal obstacle is presented by the considerable heterogeneity in vitamin D dosages utilized across various clinical trials, which varies from negligible supplemental amounts to intensive regimens. This variability impedes the establishment of the optimal dosage required to influence adipose tissue inflammation, augment insulin sensitivity, or enhance muscular and neurovascular functionalities. Initial vitamin D insufficiency, disparities in adiposity, and fluctuations in bioavailability among individuals with obesity further obfuscate the interpretation of dosing effects (121, 122). Exercise protocols exhibit significant variability across different studies, encompassing interventions that range from low-intensity aerobic training and moderate continuous exercise to resistance training programs and high-intensity interval training. This heterogeneity in exercise modality, frequency, and duration engenders inconsistent metabolic and neuromuscular adaptations, thereby impeding the identification of a standardized training paradigm that optimally may enhance synergistic effects when combined with vitamin D (89). Furthermore, the characteristics of participants are markedly diverse across various trials. Variations in factors such as age, sex, ethnicity, the severity of obesity, metabolic status, comorbid conditions, and habitual levels of physical activity significantly influence both vitamin D metabolism and the responsiveness to exercise, thereby diminishing the comparability among study populations and limiting the applicability of findings to clinical settings (67). Another significant constraint pertains to the relatively brief duration of the majority of interventions, which are frequently confined to a span of 8–16 weeks. Such temporal limits may prove inadequate for accurately assessing the long-term ramifications on neurovascular–muscle axis functionality, adipose tissue remodeling, cognitive performance, renal biomarkers, or enduring metabolic enhancements. Longitudinal investigations extending beyond 6 months are notably rare, thereby hindering definitive assertions regarding chronic adaptations or the longevity of treatment effects (123). Furthermore, despite the robust mechanistic associations between both vitamin D and physical activity with neurocognitive well-being, only a limited number of clinical trials have incorporated cognitive or neurovascular outcomes, thereby leaving a considerable translational void. Mitigating these limitations through the implementation of standardized dosing protocols, uniform training modalities, clearly delineated populations, and extended intervention durations will be imperative for elucidating the therapeutic potential of the interactions between vitamin D and exercise in individuals with overweight or obesity. An additional consideration that warrants further investigation is the optimal sequencing and timing of vitamin D supplementation relative to exercise initiation. Although the present review focuses primarily on concurrent exposure, it remains unclear whether correcting vitamin D deficiency prior to initiating structured exercise, versus initiating supplementation simultaneously with training, differentially influences metabolic, muscular, or neurovascular adaptations. From a physiological perspective, restoring adequate vitamin D status before exercise could theoretically optimize calcium handling, mitochondrial function, and vitamin D receptor–mediated signaling, thereby creating a more permissive environment for training adaptations. Conversely, exercise itself may enhance vitamin D metabolism and tissue responsiveness, suggesting potential bidirectional interaction. However, current clinical trials rarely stratify participants based on baseline vitamin D status at exercise onset or directly compare sequencing strategies, limiting causal inference. Well-powered, deficiency-stratified randomized studies explicitly designed to evaluate timing and combination effects are needed to determine whether specific sequencing approaches confer advantages beyond concurrent implementation.

Evidence from clinical studies and systematic analyses indicates that long-term or moderately high doses of supplemental vitamin D can increase the risk of hypercalcemia in a small subset of individuals. For example, a recent meta-analysis found that supplemental vitamin D doses of 3200–4000 IU per day were associated with a significantly increased risk of hypercalcemia compared with placebo, despite this being an occasional adverse event in most participants (124).

Additionally, randomized data show that hypercalcemia occurs more frequently with higher doses of vitamin D, although it is generally rare, mild, and transient in healthy adult populations (125).

Separate reports also caution that excessive high-dose vitamin D supplementation can precipitate severe hypercalcemia in clinical contexts such as granulomatous disorders, where dysregulated vitamin D metabolism increases susceptibility to hypercalcemia (126). Taken together, these data support the importance of discussing hypercalcemia risk, particularly when considering high-dose supplementation protocols, and reinforce the need for clinical monitoring of serum calcium and 25-hydroxyvitamin D levels in studies employing supraphysiological dosing.

## 8 Integrative mechanistic model: the neurovascular–muscle axis

The neurovascular–muscle axis provides a conceptual framework to integrate the complementary actions of vitamin D and physical exercise across neural, vascular, muscular, and metabolic systems in obesity. Mechanistically, vitamin D signaling via the vitamin D receptor may enhance calcium handling, mitochondrial function, endothelial integrity, and anti-inflammatory tone, thereby creating a biological environment that may facilitate exercise-induced adaptations. Exercise, in turn, acts as the primary physiological stimulus driving improvements in insulin sensitivity, muscle remodeling, vascular perfusion, and neurotrophic signaling. While preclinical data suggest that these pathways may interact synergistically at molecular and cellular levels, available human evidence more consistently supports additive or permissive effects, in which adequate vitamin D status may enhance tissue responsiveness to exercise rather than independently driving superior outcomes. Accordingly, the neurovascular–muscle axis should be viewed as a testable mechanistic model that explains how vitamin D may modulate exercise efficacy, rather than as definitive proof of clinical synergy (127, 128). Vitamin D promotes cellular responsiveness to exercise through the activation of the VDR within endothelial cells, skeletal muscle fibers, neurons, and adipocytes (129). Concurrently, exercise elevates VDR expression, mitochondrial functionality, and perfusion capacity, thereby intensifying vitamin D-dependent biological pathways (130, 131). At the molecular dimension, the activation of the VDR facilitates mitochondrial biogenesis, augments antioxidant defenses, regulates calcium handling, and may enhance insulin signaling pathways (132), whereas physical exercise stimulates complementary metabolic pathways via PGC-1α, AMPK, and mTOR signaling cascades (133). These integrative mechanisms mitigate chronic inflammation by inhibiting NF-κB and diminishing the levels of TNF-α and IL-6, concurrently elevating the concentrations of IL-10 and adiponectin (134, 135). Enhanced endothelial and muscular functionality promotes the delivery of oxygen, the uptake of glucose, and the oxidation of fatty acids, thereby contributing to the alleviation of adipose dysfunction and the enhancement of hepatic and renal metabolic profiles (136, 137). Upstream enhancements in vascular and muscular vitality morph into downstream neurocognitive boons, showcasing amplified cerebral blood flow, elevated BDNF expression, and diminished neuroinflammation, all of which bolster synaptic plasticity and executive prowess (138, 139). Within these interwoven pathways, vitamin D and exercise unite on a singular mechanistic journey that intertwines transcriptional, metabolic, and behavioral outcomes. Collectively, this cohesive model weaves together molecular, and behavioral ramifications. The neurovascular–muscle nexus presents an intricate scaffold for grasping the multi-organ advantages of vitamin D–exercise combination and lays down a mechanistic bedrock for precision lifestyle strategies tailored to individuals with overweight and obesity.

## 9 Modifiers of vitamin D–exercise responsiveness in obesity

The physiological and clinical effects of combined vitamin D supplementation and exercise are shaped by substantial interindividual variability, which is particularly pronounced in populations with overweight and obesity. Genetic, hormonal, metabolic, nutritional, and environmental modifiers critically influence vitamin D bioavailability, receptor signaling, and downstream exercise adaptations, and therefore must be considered when interpreting outcomes within the neurovascular–muscle axis.

### 9.1 Genetic determinants and VDR polymorphisms

Polymorphisms, genetic variants present in at least 1% of the population, can influence brain function and susceptibility to neurocognitive disorders. Variants in regulatory regions of genes can alter gene expression levels, affecting protein abundance and activity in the brain. For example, changes in promoter regions can modify mRNA transcription, while 3′ untranslated region (UTR) variants can impact mRNA stability and translation efficiency, potentially altering neuronal signaling pathways (140). Some polymorphisms occur within coding regions, leading to changes in protein structure or function. The *Fok*I polymorphism in exon 2 of the vitamin D receptor (VDR) gene illustrates this: a single nucleotide change alters the start codon, producing a shorter, more transcriptionally active protein. Such alterations can affect neurocognitive processes that rely on vitamin D signaling, including neuronal differentiation, synaptic plasticity, and neuroprotection (141, 142). Association studies linking polymorphisms to brain disorders suggest that certain genetic variants may increase susceptibility to conditions such as Alzheimer’s disease, cognitive decline, and other neurodegenerative or neurodevelopmental disorders. However, functional effects of many polymorphisms remain unclear, and their influence can be gene- and cell type-specific, highlighting the complexity of genetic contributions to neurocognition (143).

Recent sequencing efforts have identified additional VDR variants, including promoter (Cdx2) and 3′ UTR polymorphisms, which may further modulate gene expression in brain cells. Understanding these variants and their functional impacts is crucial for elucidating the genetic basis of cognitive impairment and for developing targeted preventive or therapeutic strategies (143). Polymorphisms in the vitamin D receptor (VDR) gene (e.g., *Fok*I, *Bsm*I, *Apa*I, *Taq*I) have been shown to alter VDR expression, ligand binding affinity, and transcriptional activity, thereby influencing individual responsiveness to vitamin D supplementation (144–146). Emerging evidence suggests that these variants may modify muscle strength, insulin sensitivity, inflammatory tone, and neurocognitive outcomes, as well as the magnitude of exercise-induced adaptations. In obesity, where VDR signaling may already be blunted by chronic inflammation and altered adipokine profiles, genetic variability likely contributes to heterogeneous clinical responses and may partially explain inconsistent findings across trials (147). Several common VDR polymorphisms, particularly *Fok*I (rs2228570), *Bsm*I (rs1544410), *Apa*I (rs7975232), and *Taq*I (rs731236), have been associated with measurable differences in vitamin D signaling and clinical outcomes (148). The *Fok*I polymorphism is functionally relevant because it alters the translation initiation site of the receptor, producing either a shorter (424 amino acids) or longer (427 amino acids) VDR protein. Experimental data suggest that the shorter isoform exhibits approximately 1.5–1.7-fold greater transcriptional activity, which may translate into enhanced downstream gene activation in response to 1,25(OH)2D. This structural difference provides a plausible biological basis for variability in supplementation response (149).

Population studies report that allele frequencies for these polymorphisms vary substantially by ethnicity; for example, the F allele of FokI ranges from approximately 40%–60% in European populations but may differ in Asian and Middle Eastern cohorts. Clinical associations are generally modest but consistent in direction. Meta-analyses have shown that certain VDR variants are associated with 2%–5% differences in bone mineral density and modest alterations in fracture risk. In metabolic contexts, some studies report that individuals carrying specific BsmI or TaqI genotypes exhibit differential changes in insulin resistance indices or inflammatory markers following supplementation, though effect sizes are small (often explaining <5% of outcome variance) (149). Importantly, while several trials demonstrate genotype-dependent differences in serum 25(OH)D increments after supplementation, typically on the order of 5–15 nmol/L between genotype groups, these findings are not universally replicated. Heterogeneity in baseline deficiency status, dosing regimens (ranging from 800 IU/day to >4000 IU/day), duration, and population characteristics limits direct comparison. Moreover, most interventional studies were not originally powered for genotype-stratified analyses, increasing the likelihood of under- or overestimation of true effects (149). A systematic review in this field emphasizes that although VDR polymorphisms contribute to interindividual variability, they account for only a fraction of the observed heterogeneity in vitamin D responsiveness. Gene–gene interactions (e.g., with GC or CYP2R1 variants), adiposity-related sequestration, inflammatory status, and lifestyle factors likely play equal or greater roles (149).

### 9.2 Dosing, formulation, and bioavailability of vitamin D in obesity

Vitamin D is a fat-soluble secosteroid synthesized in the skin following sunlight exposure. This process begins when ultraviolet B (UV-B) rays, specifically in the 280–310 nm wavelength range, trigger a photochemical reaction in 7-dehydrocholesterol (pro-vitamin D) present in the skin (150). The initial hydroxylation of vitamin D occurs at carbon atom-25 (C-25) in the liver, where the enzyme vitamin D-25-hydroxylase (CYP2R1), a cytochrome P450-like enzyme, converts cholecalciferol into 25-hydroxyvitamin D [25(OH)D]. This compound has a half-life of approximately 2–3 weeks. The final hydroxylation step takes place in the renal proximal convoluted tubules, where the enzyme vitamin D-1-hydroxylase (CYP27B1) converts 25(OH)D into 1,25-dihydroxycholecalciferol (calcitriol), the biologically active form of vitamin D. Calcitriol has a much shorter half-life of about 6–8 h (150). Additionally, both 25(OH)D and calcitriol can undergo further hydroxylation by the vitamin D-24-hydroxylase (CYP24A1), found in various human tissues, leading to the formation of 24,25-dihydroxyvitamin D3 [24,25(OH)2D3] and 1,24,25-trihydroxyvitamin D3 [1,24,25(OH)3D3]. These metabolites do not play significant biological roles and are excreted through bile, feces, and urine (151). Numerous studies have assessed serum 25(OH)D levels following vitamin D supplementation across various age groups, including children, adolescents, adults, and the elderly. There is agreement that the rise in serum 25(OH)D is contingent on the initial vitamin D status; individuals who are vitamin D deficient show the most significant increases in 25(OH)D with supplementation, while those with higher baseline levels experience progressively smaller changes. Additionally, the quantity of vitamin D required to attain a specific level, such as 20 ng/mL, varies based on factors like age, body weight, gene polymorphisms, and the methods used for measurement (152, 153).

Vitamin D dosing strategies represent a critical determinant of efficacy in individuals with obesity, in whom adipose tissue sequestration reduces circulating and bioactive 25(OH)D levels. Several pathophysiological mechanisms have been proposed to clarify this relationship. Excess body fat can act as a reservoir for vitamin D, affecting the dynamics between stored vitamin D and circulating levels. Furthermore, obesity may be linked to decreased dietary intake of vitamin D, less outdoor physical activity leading to limited sunlight exposure, impaired hydroxylation in adipose tissue, and changes in vitamin D receptor function (154, 155). Individuals with obesity, especially older adults, frequently exhibit low levels of 25(OH)D, which show an inverse correlation with body mass index and fatness. This deficiency negatively impacts bone and muscle health, leading to a higher risk of an osteo-sarcopenic phenotype with obesity. The prevalence of vitamin D deficiency is significantly higher, by 35% in individuals with obesity and 24% in overweight, compared to those of normal weight. Since adipose tissue acts as a reservoir for vitamin D, the low circulating levels may indicate its accumulation in fat stores. However, the specific mechanisms by which vitamin D is stored in adipose tissue remain unclear (156, 157).

In bioavailability point pf view, cholecalciferol (vitamin D3) suggests superior bioavailability and potency compared with ergocalciferol (vitamin D2), particularly in the context of high adiposity. Higher or weight-adjusted dosing regimens may therefore be necessary to achieve biologically effective concentrations capable of modulating muscle, vascular, and neural pathways. Failure to account for these pharmacokinetic considerations may underestimate the true modulatory role of vitamin D in exercise interventions. A study measuring blood levels of vitamin D3 24 h after whole-body irradiation revealed that the increase in vitamin D3 was 57% less in participants with obesity compared to their non-obesity counterparts (158). Importantly, there were no significant differences in the levels of the vitamin D3 precursor, 7-dehydrocholesterol, in the skin of both groups, nor in its conversion to previtamin D3 following irradiation *in vitro* (158). After receiving an oral dose of 50,000 IU (1.25 mg) of vitamin D2, body mass index (BMI) showed an inverse correlation with serum vitamin D3 levels post-irradiation (*r* = −0.55, *P* = 0.003) and with peak serum vitamin D2 levels following intake (*r* = −0.56, *P* = 0.007). they concluded that Vitamin D insufficiency in individuals with obesity is likely attributable to reduced bioavailability of vitamin D3 from both sun exposure and dietary sources, as it becomes stored in body fat compartments (158).

### 9.3 Sex, hormonal status, and life stage

Sex-specific differences in vitamin D metabolism, muscle plasticity, and inflammatory regulation further influence responses to combined interventions. Studies have examined sex-related differences in serum 25OH vitamin D (25OHD) levels across various body mass index (BMI) categories and to determine if any observed differences could be attributed to variations in body composition between sexes. In one study, a total of 500 participants were enrolled, comprising 250 males (average age 37.4 ± 11.8 years) and 250 females (average age 36.6 ± 11.8 years) (159). The study findings indicated that females had lower 25OHD concentrations than their male counterparts. Additionally, female subjects with vitamin D deficiency exhibited a higher percentage of fat mass (FM) compared to males with the same deficiency. In both sexes, 25OHD levels showed an inverse relationship with BMI, percentage of fat mass, and extracellular water (ECW). Conversely, 25OHD concentrations were directly related to reactance (Xc), total body water (TBW), and intracellular water (ICW). Notably, a positive correlation between resistance (R) and 25OHD concentrations was observed exclusively in males. Females exhibited lower 25OHD concentrations than males with comparable BMI, likely attributed to the higher fat mass in females. Generally, men tend to have 10%–15% less fat content than women at the same BMI (159).

Another study assessed seasonal variations and the prevalence of vitamin D deficiency across various body mass index (BMI), sex, and age groups (160). Findings revealed a significant decline in serum 25(OH)D3 levels with increasing BMI for both sexes and across two age categories (under 50 years and 50 years or older). Interestingly, both 25(OH)D3 and serum 1,25(OH)2D3 levels were negatively correlated with BMI. The greatest seasonal fluctuations in serum 25(OH)D3 were observed in younger man with non-obesity. The prevalence of vitamin D deficiency was notably high among individuals with a BMI of 40 or greater, reaching 32% in women and 46% in men. In conclusion, the levels of 25(OH)D3, along with their seasonal variations and the prevalence of vitamin D deficiency, are influenced by BMI and age independently. The study indicates that approximately one in three women and one in two men with a BMI of 40 or higher are vitamin D deficient (160). Upon stratifying participants by sex and BMI categories, they found that 25OHD levels were significantly higher in males across all BMI classifications and declined with increasing BMI. Females with vitamin D deficiency exhibited a higher percentage of fat mass (FM) compared to their male counterparts. Additionally, 25OHD levels were inversely correlated with FM% in both sexes. A multiple regression analysis revealed that sex, FM%, and BMI were significant predictors of 25OHD concentrations. In summary, our findings indicate that 25OHD levels were consistently lower in females than in males (160). Estrogen status, menopause, androgen levels, and age-related endocrine changes modify calcium handling, mitochondrial function, and VDR expression, thereby shaping exercise responsiveness. Postmenopausal women and older adults with obesity may therefore exhibit distinct adaptations compared with younger or male cohorts, underscoring the need for sex- and life stage–specific analyses in future trials.

### 9.4 Micronutrient interactions (calcium, magnesium, vitamin K)

Although the manuscript focuses primarily on the roles of vitamin D and exercise, interactions among micronutrients such as calcium, magnesium, and vitamin K may modulate vitamin D metabolism, physiological effects, and related health outcomes and warrant brief consideration. Calcium is the principal downstream effector of vitamin D–mediated intestinal absorption and systemic mineral homeostasis. Vitamin D may enhance active transcellular calcium transport via upregulation of TRPV6, calbindin, and related transport proteins, thereby influencing skeletal integrity, neuromuscular function, and potentially cardiometabolic regulation. In obesity, altered calcium metabolism and secondary hyperparathyroidism may further complicate vitamin D physiology, and combined calcium–vitamin D supplementation has been shown in some trials to modestly influence adiposity and metabolic indices, although effects remain heterogeneous (161). Calcium and vitamin D have a well-established functional relationship, as vitamin D may enhance intestinal calcium absorption and contributes to systemic calcium homeostasis, which may influence musculoskeletal and cardiometabolic traits relevant to obesity and exercise adaptation. Systematically reviewing populations with obesity, calcium and magnesium status are commonly interrelated with vitamin D status, and deficiencies of these minerals have been associated with adverse metabolic markers, including insulin resistance and adiposity indices (162). Magnesium also has critical interactions with vitamin D and calcium: it acts as a cofactor in vitamin D activation and transport, and imbalances in dietary calcium:magnesium ratios may influence chronic disease risk and nutrient metabolism (163). In addition, evidence in bone health contexts highlights that vitamin K and vitamin D act synergistically through vitamin K-dependent proteins such as osteocalcin to regulate calcium deposition and skeletal integrity. These interrelationships suggest that future research on vitamin D and exercise should consider concurrent micronutrient status and interactions to more comprehensively characterize physiological responses and optimize translational relevance (164).

### 9.5 Gut microbiota

Emerging evidence indicates that the gut microbiota may function as an upstream regulator and bidirectional modifier of vitamin D metabolism and signaling, thereby influencing systemic metabolic, inflammatory, and neurovascular processes relevant to obesity (165). Although this interaction has been more extensively studied in the context of inflammatory bowel disease and autoimmune disorders, accumulating data suggest that microbiota–vitamin D axis may also modulate metabolic homeostasis and tissue responsiveness in individuals with excess adiposity (165). Vitamin D absorption occurs primarily in the small intestine and is dependent on micelle formation, bile acid metabolism, and epithelial integrity. The gut microbiota contributes to bile acid transformation and regulates intestinal permeability through modulation of tight junction proteins and mucosal immune tone (166). Dysbiosis–frequently observed in obesity, is associated with impaired barrier function, endotoxemia, and chronic low-grade inflammation. Such alterations may indirectly influence vitamin D bioavailability and downstream vitamin D receptor (VDR) activation. Conversely, adequate vitamin D status has been shown to support epithelial barrier integrity, enhance antimicrobial peptide expression (e.g., cathelicidin), and reduce pro-inflammatory signaling within the intestinal mucosa, suggesting a reciprocal regulatory loop between vitamin D signaling and microbial ecology (167). At the molecular level, the VDR is expressed in intestinal epithelial cells and immune cells residing within the gut-associated lymphoid tissue (168). Experimental models demonstrate that VDR signaling shapes microbial composition, while VDR deficiency is associated with altered microbial diversity, increased intestinal permeability, and exaggerated inflammatory responses. These findings imply that host vitamin D signaling capacity may influence microbial stability, which in turn modulates systemic inflammation and metabolic regulation. In obesity, where both VDR expression and microbial diversity may be perturbed, this interaction could contribute to variability in metabolic responsiveness to vitamin D supplementation (168, 169).

Short-chain fatty acids (SCFAs), particularly butyrate, acetate, and propionate, represent another potential mechanistic interface. SCFAs produced by commensal bacteria exert anti-inflammatory effects, enhance insulin sensitivity, and influence mitochondrial function. Exercise is known to increase microbial diversity and SCFA-producing taxa, thereby improving metabolic flexibility and reducing adipose inflammation (170). Vitamin D signaling intersects with similar inflammatory and metabolic pathways, including NF-κB suppression and AMPK activation. Although direct evidence for a three-way interaction among microbiota, vitamin D, and exercise in obesity remains limited, overlapping mechanistic pathways suggest that microbiota composition may modify the magnitude of both vitamin D– and exercise-induced adaptations (171). Human studies examining associations between circulating 25(OH)D concentrations and microbial diversity report correlations between higher vitamin D status and increased α-diversity, as well as enrichment of butyrate-producing taxa (172, 173). However, these findings are largely observational and influenced by confounding variables such as diet, adiposity, and physical activity. Interventional trials investigating vitamin D supplementation show variable effects on microbiota composition, with changes often modest and context-dependent, particularly in individuals without severe deficiency (173). On the other hand, the gut microbiota contributes to several of processes and may therefore influence vitamin D bioavailability.

First, intestinal microbes participate in bile acid metabolism through deconjugation and biotransformation reactions. Because bile acids facilitate micelle formation required for fat-soluble vitamin absorption, microbial alterations in bile acid pools may indirectly modify vitamin D uptake efficiency. Dysbiosis-associated disruptions in bile acid signaling, frequently observed in obesity, may impair micellar solubilization and reduce intestinal absorption of vitamin D (174).

Second, the microbiota plays a central role in maintaining epithelial barrier integrity. Commensal bacteria regulate tight junction protein expression and mucosal immune tone, while dysbiosis is associated with increased intestinal permeability and endotoxemia. Impaired barrier function may alter transcellular transport processes and interfere with nutrient absorption, including fat-soluble vitamins. Experimental models suggest that vitamin D deficiency itself worsens epithelial dysfunction, creating a potential feedback loop in which dysbiosis reduces vitamin D absorption, and low vitamin D status further destabilizes barrier integrity (175).

Third, microbial metabolites, particularly SCFAs, influence intestinal epithelial energy metabolism and transporter expression. Butyrate, for example, supports enterocyte function and may enhance absorptive capacity. Although direct human evidence linking SCFA production to enhanced vitamin D absorption remains limited, mechanistic overlap suggests that microbial metabolic activity could modulate intestinal efficiency for fat-soluble micronutrients (176).

Taken together, current evidence supports a bidirectional relationship in which (1) gut microbiota may influence vitamin D absorption, metabolism, and tissue responsiveness, and (2) vitamin D signaling may modulate microbial composition and intestinal immune homeostasis. Within the neurovascular–muscle axis framework, the microbiota may therefore act as a modulatory node linking intestinal integrity, systemic inflammation, metabolic flexibility, and neuroinflammatory tone. Nevertheless, causal evidence demonstrating that microbiota-mediated mechanisms significantly alter clinical responsiveness to combined vitamin D supplementation and exercise in populations with obesity remains insufficient. Future studies incorporating microbiome profiling, vitamin D status stratification, and standardized exercise protocols will be required to clarify whether the gut microbiota meaningfully modifies therapeutic outcomes within this integrative model.

### 9.6 Comorbidities such as depression

In considering combined vitamin D supplementation and exercise interventions in individuals with obesity, it is increasingly recognized that comorbid conditions such as depression, metabolic syndrome, and chronic inflammation may act as biological and behavioral modifiers of treatment response, rather than merely outcomes that improve with intervention. Obesity and depression share complex and bidirectional pathophysiological links, with epidemiological studies demonstrating that depressive symptoms are more prevalent in individuals with higher adiposity, chronic systemic inflammation, and insulin resistance (177). This comorbidity cluster is associated with dysregulated hypothalamic–pituitary–adrenal (HPA) axis activity, altered cytokine profiles (e.g., elevated IL-6, TNF-α), and impaired neuroendocrine function, all of which are known to influence muscle metabolism, energy balance, and neuroplasticity, key pathways targeted by both exercise and vitamin D signaling (178, 179). From a behavioral perspective, depressive symptoms are associated with reduced motivation, fatigue, and decreased engagement in physical activity, which can limit adherence to exercise prescriptions and reduce the physiological benefits typically observed in healthy cohorts (180). This is particularly relevant in obesity, where exercise adherence is already challenged by musculoskeletal discomfort and low baseline fitness. Lower physical activity levels have been linked to reduced improvements in insulin sensitivity, cardiorespiratory fitness, and body composition following structured exercise regimens, indicating that baseline psychological health may shape the extent of responsiveness. Furthermore, chronic stress and mood disorders have been shown to influence appetite regulation, sleep quality, and rewards-related eating behaviors, all of which can counteract weight management efforts and metabolic adaptations expected from lifestyle interventions (181). Biologically, depression and obesity share inflammatory signatures that intersect with vitamin D metabolism. Several observational and interventional studies report that lower serum 25-hydroxyvitamin D concentrations are associated with greater depressive symptom severity and heightened inflammatory markers in individuals with excess adiposity, suggesting that inflammatory burden may reduce bioavailability or receptor responsiveness to vitamin D (182). Although vitamin D has immunomodulatory effects, the presence of chronic inflammation may blunt these responses, potentially requiring higher or more sustained doses for comparable effects seen in individuals without inflammatory comorbidity. This interplay may also affect exercise adaptations, as inflammatory cytokines can impair muscle protein synthesis, mitochondrial function, and neurotrophic factor expression, all of which contribute to exercise-induced improvements in metabolic and cognitive outcomes (183). Meta-analytical evidence also supports the notion that baseline health status modifies the magnitude of intervention effects: vitamin D supplementation trials often report greater changes in depressive symptoms in participants with existing deficiencies or clinical depressive states compared to non-deficient or asymptomatic cohorts (184). Likewise, exercise interventions demonstrate heterogeneity in outcomes based on baseline psychological distress, with individuals exhibiting higher depressive symptoms prior to intervention sometimes showing attenuated physiological adaptations, reduced exercise adherence, and smaller gains in cardiorespiratory fitness (184). Collectively, this evidence suggests that comorbid depression and related psychosocial factors should be treated as modifiers of response when interpreting the efficacy of combined vitamin D and exercise strategies in populations with obesity, highlighting the need for stratified analysis in future randomized trials and for the development of tailored interventions that incorporate psychological support alongside metabolic and lifestyle therapies.

## 10 Observational and randomized evidence: interaction effects and subgroup-specific responses

Evidence from epidemiological analyses and randomized trials provides complementary insights into the relationship between vitamin D status, physical activity, and aging-related outcomes. Observational datasets offer valuable information about real-world associations and potential synergistic interactions, whereas randomized interventions help clarify whether such associations translate into measurable clinical benefits. A population-based analysis using National Health and Nutrition Examination Survey (NHANES) data evaluated 18,738 participants to investigate the independent and joint associations between serum 25-hydroxyvitamin D [25(OH)D] levels, physical activity, and phenotypic age acceleration. Biological aging was assessed using the Phenotypic Age framework, with multivariable models adjusting for socioeconomic factors, comorbidities, lifestyle variables, and anthropometric measures such as body mass index (BMI). Higher vitamin D concentrations and adequate physical activity were each independently associated with reduced likelihood of accelerated aging; however, combined exposure produced a stronger protective association than either factor alone, suggesting interaction rather than simple additive effects. The combined effect appeared particularly pronounced in younger and middle-aged adults (≤65 years), where a significant multiplicative interaction between vitamin D status and activity level was observed. Participants exceeding approximately 80 nmol/L of 25(OH)D while maintaining adequate physical activity exhibited lower phenotypic age estimates, corresponding to a measurable reduction in biological aging relative to chronological age. Population attributable fraction analyses further suggested that a substantial proportion of accelerated aging cases could theoretically be avoided if both low vitamin D status and physical inactivity were addressed simultaneously (185). Subgroup analyses in this observational study indicated heterogeneity in response patterns. Age-stratified models demonstrated stronger associations in adults below older age thresholds, implying diminishing marginal benefit in later life. Sex and BMI were included as covariates in adjusted models, reflecting their influence on vitamin D metabolism and aging phenotypes; although the main findings remained consistent after adjustment, the study emphasized that body composition and metabolic health may modulate responsiveness. Additionally, non-linear dose–response analyses suggested threshold effects for vitamin D, indicating that benefits may plateau beyond certain concentrations. These results reinforce the concept that biological aging trajectories are influenced by the interaction of lifestyle exposures rather than isolated behaviors. Statistical modeling indicated that the interaction between these variables exceeded simple additive effects, implying a potential synergistic relationship. Participants meeting thresholds for both adequate vitamin D levels and recommended physical activity demonstrated more favorable biological age estimates and reduced prevalence of accelerated aging phenotypes. These findings highlight the possibility that micronutrient sufficiency and habitual movement jointly contribute to healthier aging trajectories through shared mechanisms such as inflammation regulation, oxidative stress modulation, and maintenance of metabolic resilience (185).

In contrast, randomized evidence from the DO-HEALTH trial offers a more nuanced interpretation of these associations. This large factorial randomized clinical trial enrolled over 2,100 community-dwelling older adults across Europe (mean age approximately 75 years) and examined daily vitamin D3 supplementation (2000 IU), omega-3 fatty acids, and a structured strength-training exercise program, alone and in combination, over 3 years. Despite strong biological plausibility, the interventions did not significantly improve primary clinical outcomes such as blood pressure, cognitive performance, physical function, fractures, or infection incidence overall. Predefined subgroup analyses, however, revealed several differential effects that highlight population heterogeneity. For example, vitamin D supplementation demonstrated a significant interaction with sex for systolic blood pressure, with men experiencing modest reductions whereas women did not show benefit. Age-stratified analyses indicated that younger participants within the older cohort (70–74 years) had fewer infections when receiving vitamin D compared with placebo, whereas effects were absent or less consistent in older age groups. Baseline vitamin D deficiency (<20 ng/mL) did not significantly modify treatment effects for blood pressure, suggesting that supplementation benefits may not be restricted solely to deficient individuals. Additional exploratory analyses examined BMI, prior health conditions, and baseline activity levels, though no consistent differential effects emerged across these factors (186). Together, these findings illustrate the complexity of translating observational vitamin D-exercise combined effect into clinical outcomes. Epidemiological evidence suggests that optimal vitamin D status combined with regular physical activity may be associated with slower biological aging, particularly among younger or metabolically resilient individuals. However, randomized trials in relatively healthy older adults demonstrate limited overall clinical benefit, with subgroup analyses suggesting that responsiveness may vary according to sex, age strata, and baseline physiological reserve. The divergence between observational and experimental data underscores the likelihood that synergistic effects depend on threshold conditions, including baseline vitamin D status, metabolic health, and intervention timing across the lifespan. Future trials targeting individuals with lower baseline nutrient status, higher BMI-related metabolic risk, or early functional decline may be better positioned to detect clinically meaningful combined effect.

## 11 Conclusions and future directions

Obesity is characterized by tightly interconnected disturbances in skeletal muscle metabolism, vascular function, and neurocognitive health that cannot be fully explained when these systems are considered in isolation. In comparison to the recent 2025 literature on the interactions between vitamin D and exercise, this review offers a comprehensive synthesis that both extends and integrates these findings within a broader context of obesity-related health complications. The studies by Chen et al. (15), Peng et al. (16), Jawed et al. (17), and Farina et al. (49) have made significant strides in demonstrating the neuroprotective synergy of vitamin D and exercise, highlighting their impact on muscle health, neurocognitive function, and metabolic outcomes. However, our review further builds on these studies by adopting a more holistic framework, the neurovascular-muscle axis, that emphasizes the interconnected nature of neural, vascular, and muscular health in obesity. While previous works have primarily focused on isolated mechanisms, such as the role of vitamin D in muscle function or neuroprotection, our review integrates these aspects into a cohesive understanding of how vitamin D and exercise may work synergistically to influence systemic metabolic health, reduce adipose inflammation, and improve insulin sensitivity.

In particular, our review expands on the findings of Peng et al. (16) by exploring the nuanced effects of vitamin D supplementation in enhancing exercise-induced weight loss, particularly in populations with vitamin D deficiency, and how this interplay can be leveraged to improve metabolic outcomes. Moreover, while Chen et al. (15) focus on the neuroprotective benefits of this combination, we go further by discussing the mechanisms that underlie neurovascular coupling and cognitive health in the context of obesity, which remains a critical but often overlooked aspect. The inclusion of Jawed et al. (17) and Farina et al. (49) in the discussion enriches our understanding of muscle health and neuroprotection, particularly as we address how exercise-induced myokines and vitamin D receptor signaling can enhance muscle function and cognitive performance. By synthesizing these recent studies within the framework of a neurovascular–muscle axis, we offer a novel perspective that both connects and extends the current literature, emphasizing the importance of a multifaceted approach. This review not only underscores the complementary roles of vitamin D and exercise but also highlights the gaps in understanding, particularly regarding long-term, population-specific responses to these interventions. Thus, while the 2025 studies contribute invaluable insights into the therapeutic potential of combining vitamin D and exercise, this review provides an innovative, integrative perspective that lays the groundwork for future clinical trials and personalized therapeutic strategies in obesity-related metabolic diseases.

In this review, we propose the neurovascular–muscle axis as a conceptual integrative framework that synthesizes emerging evidence linking neural signaling, endothelial integrity, and muscle-derived metabolic regulation in the context of obesity. Within this framework, vitamin D and physical exercise emerge as complementary modulators that converge on shared molecular pathways, including inflammatory signaling, mitochondrial biogenesis, calcium homeostasis, and neurotrophic support, to influence metabolic flexibility and systemic homeostasis. While direct experimental validation of this axis as a unified physiological system remains limited, converging findings from animal and human studies support the plausibility of bidirectional interactions among its components. Importantly, the current evidence indicates that exercise is a primary driver of metabolic and functional improvement, with vitamin D potentially acting as a permissive or amplifying factor, particularly in deficient populations. Future well-powered, longitudinal, and mechanistically integrated studies are needed to directly test this axis, clarify causality, and determine whether targeting neurovascular–muscle axis can enhance personalized preventive and therapeutic strategies for obesity-related metabolic and cognitive dysfunction. The collective evidence from both preclinical and clinical studies suggests that exercise consistently improves metabolic, inflammatory, and insulin-related outcomes, while the additional benefits of vitamin D supplementation are more variable and appear to depend on baseline vitamin D status, dosage, and type of exercise. In animal models, combining exercise (swimming, voluntary activity, or aerobic training) with relatively high doses of vitamin D (ranging from 500 IU/kg to 50,000 IU/week) often produced additive or synergistic improvements in body weight, adiposity, lipid profiles, inflammatory markers, and insulin sensitivity, with mechanistic evidence pointing to pathways such as FATP4/TLR4 and AMPK/PGC-1α/UCP1. In human studies, aerobic and resistance training interventions combined with moderate to high vitamin D doses (1,200 IU/day to 50,000 IU/week) generally improved adiposity, lipid profiles, and glycemic control, particularly in individuals with vitamin D deficiency; however, several studies reported that vitamin D provided minimal additional benefit beyond exercise alone, especially in otherwise healthy or non-deficient participants. Overall, the current evidence supports exercise as the primary driver of metabolic improvements, while vitamin D supplementation may enhance outcomes primarily in deficient populations and at adequate dosages. High-intensity or aerobic exercise appears most effective for improving insulin sensitivity and metabolic homeostasis, and supplementation regimens of ≥2,000 IU/day up to 50,000 IU/week are commonly used in studies showing additive effects. Although promising, the number of human studies remains limited, and further well-powered clinical trials are required to define optimal dosing, exercise type, and the populations most likely to benefit from combined vitamin D and exercise interventions.

As summarized in Table 3, exercise-induced metabolic improvements are consistently supported in obesity populations, whereas additive effects of vitamin D supplementation remain context-dependent and, in many mechanistic domains, mechanistically inferred rather than definitively established.

**TABLE 3:** Evidence classification of vitamin D–exercise interactions based exclusively on intervention studies cited in this manuscript.

Interaction domain	Study evidence	What the data actually show	Evidence classification in obesity context
**Exercise → improved insulin sensitivity**	(69, 70, 74, 77, 113)	Exercise consistently improves glucose tolerance, insulin, HOMA-IR, or glycemic indices in humans with overweight/obesity	Empirically supported in humans with obesity
**Exercise → improved adiposity and lipid profile**	(70, 74, 77, 113)	Aerobic, HIIT, and circuit training reduce body weight, fat mass, TG, LDL, and improve HDL	Empirically supported in humans with obesity
**Vitamin D alone → metabolic improvement**	(70, 74, 88, 91)	Vitamin D increases serum 25OHD; metabolic effects alone are minimal or inconsistent	Limited independent effect in obesity humans
**Vitamin D + Exercise → additive metabolic benefit**	(69, 73, 77, 113)	Some studies report greater improvements with combined intervention (especially in vitamin D–deficient populations); others show no additional benefit beyond exercise	Supported in related human contexts (deficiency-specific); not consistently confirmed
**Vitamin D + Exercise → no additional benefit**	(70, 74, 91)	Several RCTs show exercise benefits occur independently of vitamin D supplementation	Additive/synergistic effect not empirically established
**Vitamin D + Exercise → enhanced mitochondrial/molecular signaling**	(71, 72, 75) (animal models)	Combined intervention increases AMPK, PGC-1α, UCP1 expression and improves metabolic markers in rodent models	Mechanistically inferred (preclinical evidence only)
**Vitamin D + Exercise → improved bone outcomes**	(88)	Combined intervention improved BMD and PTH more than either intervention alone in vitamin D–insufficient women	Supported in deficiency-specific human context (non-metabolic domain)
**Vitamin D + Exercise → enhanced muscle performance adaptation**	(89, 91)	Modest improvements in specific performance measures (e.g., peak power, stair climbing); no consistent augmentation of strength or glucose tolerance	Limited and outcome-specific; not broadly validated

In another point of view, the collective evidence indicates that vitamin D supplementation, particularly at daily doses of 2000–4000 IU or weekly loading doses up to 16,000 IU post-bariatric surgery, combined with resistance or water-based aerobic exercise, can improve muscle function, bone health, and certain metabolic parameters in overweight, obesity, and postmenopausal populations. Resistance and multi-modal exercises appear most effective for preserving lean mass, enhancing strength, and improving balance, while supplementation alone has limited impact. However, most studies had small sample sizes, short durations, and variable adherence, and analyses often lacked robust adjustment for confounders or correction for multiple outcomes, which reduces the reliability and generalizability of effect estimates. Overall, these findings support the combined use of vitamin D and structured exercise to enhance musculoskeletal and metabolic health, but larger, longer-term trials are needed to establish optimal dosing, exercise protocols, and clinically meaningful outcomes. With respect to dosing considerations, most clinical trials in populations with overweight or obesity have employed daily vitamin D supplementation ranging from 1000 to 4000 IU, with higher doses generally reserved for deficiency correction. Individuals with obesity often require greater supplemental intake to achieve comparable circulating 25-hydroxyvitamin D concentrations, likely due to volumetric dilution and adipose sequestration; however, routine high-dose supplementation in vitamin D–replete individuals is not supported by current evidence. In line with established safety thresholds, daily intakes up to 4000 IU are generally considered tolerable for adults, although individualized dosing guided by baseline 25(OH)D levels and periodic monitoring of serum calcium is advisable. At present, supplementation strategies should prioritize correction of documented deficiency rather than pharmacological dosing intended to enhance exercise responsiveness.

Current evidence suggests that the potential additive benefits of combined vitamin D supplementation and exercise are most likely to be observed in specific subgroups, particularly individuals with documented vitamin D deficiency, older adults, those with sarcopenic obesity, and patients with metabolic comorbidities such as type 2 diabetes or non-alcoholic fatty liver disease. In these populations, vitamin D may act as a permissive or amplifying factor for exercise-induced adaptations in muscle, metabolic, and vascular function. However, adherence to structured physical activity remains a major challenge in obesity due to barriers including musculoskeletal pain, fatigue, reduced mobility, and psychosocial factors; therefore, individualized and low-impact exercise modalities (e.g., walking-based or aquatic programs) are likely to improve feasibility and long-term engagement. From a safety and economic perspective, vitamin D supplementation at commonly studied doses is low-cost and generally well tolerated when clinically monitored, whereas exercise interventions provide broad cardiometabolic benefits with favorable cost–benefit profiles. Nevertheless, important evidence gaps remain, including uncertainty regarding optimal vitamin D dosing strategies, intervention sequencing, long-term safety, and identification of responders. Addressing these gaps will require well-powered, longitudinal clinical trials specifically designed to test integrated vitamin D–exercise interventions across defined subpopulations with obesity.

Recent high-level syntheses further contextualize the vitamin D–exercise interaction and support a more conditional interpretation of combined effect. Contemporary reviews and consensus analyses indicate that exercise remains the most consistent and robust intervention for improving metabolic, musculoskeletal, and cardiometabolic outcomes across populations with obesity, whereas vitamin D supplementation shows clear benefit primarily in the context of deficiency (187). Although combined vitamin D repletion and exercise interventions may confer selective, outcome- and phenotype-dependent advantages, for example in deficient individuals, specific metabolic or musculoskeletal endpoints, or settings of impaired adaptation, current evidence does not demonstrate uniform superiority of combination strategies over exercise alone. Collectively, these findings reinforce the view that vitamin D should be considered a context-dependent adjunct rather than a primary driver of adaptation, and that claims of synergy should be framed cautiously. Future trials with adequate power, stratification by vitamin D status, and clearly defined clinical endpoints are required to determine when combined approaches meaningfully extend the benefits of exercise in obesity (16, 187). In aggregate, the available evidence supports a clinically pragmatic and conditional framework for combining vitamin D with exercise in obesity. Exercise remains the primary and most consistently effective intervention for improving insulin sensitivity, inflammatory profiles, muscle function, and cardiometabolic health, whereas vitamin D supplementation is most clearly justified in individuals with documented deficiency, sarcopenic obesity, or elevated inflammatory burden. In these populations, vitamin D repletion initiated concurrently with structured exercise training appears reasonable, with the goal of achieving physiological sufficiency rather than supraphysiologic levels, using commonly studied doses that restore circulating 25(OH)D into accepted target ranges. Current human data support the use of combined aerobic and resistance training, delivered at sufficient intensity and volume, for at least 8–12 weeks to elicit measurable metabolic and inflammatory improvements, with longer durations required for musculoskeletal and functional adaptations. While combined interventions may yield selective, endpoint- or phenotype-specific benefits, particularly in deficient individuals, superiority beyond exercise alone is not a universal finding, and claims of synergy should be interpreted cautiously. Future obesity-specific trials that stratify by baseline vitamin D status, clearly define exercise dose and modality, and focus on clinically meaningful outcomes are needed to determine when adjunctive vitamin D meaningfully may enhance exercise responsiveness. Across the reviewed studies, exercise modality appears to influence the pattern and magnitude of cardiometabolic adaptation in individuals with overweight or obesity. Aerobic training consistently suggests improvements in adiposity, lipid profile, glycemic regulation, and inflammatory markers across both clinical populations and metabolic disease models, with combined vitamin D supplementation enhancing selected metabolic and anti-inflammatory outcomes particularly in individuals with metabolic dysfunction or vitamin D deficiency.

Resistance training, in contrast, improves inflammatory regulation and physical function but does not consistently yield additional metabolic or inflammatory benefits when combined with vitamin D supplementation in generally healthy overweight adults. High-intensity interval training improves glucose tolerance and lipid metabolism; however, available evidence indicates that vitamin D supplementation does not uniformly augment these exercise-induced benefits. Circuit-based and multimodal programs appear particularly effective in metabolically compromised populations, including older adults with type 2 diabetes or central adiposity, where combined interventions produce improvements in body composition and glycemic control beyond exercise alone. Collectively, these findings suggest that aerobic or combined training modalities are most consistently associated with cardiometabolic improvement across heterogeneous obesity phenotypes, whereas resistance training primarily supports inflammatory and functional adaptations. Patients with obesity accompanied by metabolic dysfunction, insulin resistance, or vitamin D deficiency appear most likely to derive additional benefit from combined vitamin D and exercise interventions, while individuals without deficiency or with lower metabolic risk may experience improvements largely attributable to exercise alone. Adherence represents a key determinant of intervention effectiveness across the reviewed studies, many of which employed structured, supervised exercise programs and standardized supplementation protocols within defined timeframes. Short-term interventions (typically 8–16 weeks) using clearly prescribed aerobic, resistance, or multimodal training regimens appear to facilitate participant compliance and produce consistent metabolic and inflammatory improvements driven primarily by exercise. Evidence further suggests that intervention relevance may influence adherence, as individuals with vitamin D deficiency or established metabolic dysfunction demonstrate more consistent responsiveness to combined strategies than metabolically healthy participants, in whom supplementation often provides limited additional benefit. However, the predominance of short-duration trials and structured research settings limits understanding of long-term adherence and sustainability, highlighting the need for future studies incorporating extended follow-up and implementation-oriented designs. The studies discussed involve a diverse range of participants, including both animal models and human cohorts, representing various age groups, sex distributions, metabolic statuses, and degrees of obesity. In animal models (Tables 1, 2), the studies primarily focus on rodents, such as male rats and mice, aged from juvenile to adult stages, with induced obesity through high-fat or high-sucrose diets. These models are characterized by varying metabolic conditions, including insulin resistance, metabolic dysfunction, and obesity-related comorbidities such as fatty liver disease. For the human studies, a wide age range is represented, from adolescents to elderly adults, with participants in most studies being overweight or obesity, and in some cases, specifically those with type 2 diabetes, vitamin D deficiency, or other cardiometabolic risk factors. The majority of human studies include mixed-sex populations, though some focus specifically on women, particularly postmenopausal women. These individuals display a range of metabolic conditions, from relatively healthy but overweight individuals with low-grade inflammation, to those with significant metabolic dysfunction, such as insulin resistance, and conditions like non-alcoholic fatty liver disease (NAFLD). Overall, the studies reflect a broad spectrum of obesity severity and related health issues, emphasizing the need for tailored interventions based on age, sex, and metabolic health.

It is important to note that while exercise remains the dominant intervention driving metabolic and inflammatory improvements, multiple studies suggest that vitamin D supplementation provides only modest, if any, additional benefits when used in isolation. Exercise, through its direct impact on skeletal muscle function, inflammation, and metabolic regulation, is the most potent driver of systemic health improvements in individuals with obesity. However, the role of vitamin D should not be dismissed entirely. In populations with vitamin D deficiency, supplementation may serve as a complementary strategy, helping to support exercise-induced benefits, especially in terms of inflammation modulation and skeletal muscle function. We have incorporated a more critical discussion of these findings in this manuscript, acknowledging that while vitamin D is not a primary intervention for metabolic improvements, its adjunctive effects may still provide value in specific populations. This nuanced perspective highlights the need for further research to identify the contexts in which vitamin D supplementation may enhance the outcomes of exercise, particularly for individuals with insufficient vitamin D status. Although physical activity is consistently associated with improvements in metabolic and inflammatory outcomes, several barriers may limit implementation in individuals with overweight or obesity, including reduced functional capacity, heterogeneity in metabolic health, and variability in responsiveness to intervention. Evidence across the reviewed studies suggests that structured and clearly prescribed exercise programs facilitate participation and produce consistent physiological benefits, particularly when interventions are tailored to baseline health status and delivered within defined timeframes. Patient stratification also appears relevant, as individuals with vitamin D deficiency or established metabolic dysfunction demonstrate more consistent responsiveness to combined interventions than metabolically healthy participants, supporting a targeted rather than universal adjunctive role for supplementation. Additionally, modality selection may help mitigate participation barriers, with aerobic or multimodal programs demonstrating broad cardiometabolic benefits across heterogeneous obesity phenotypes, while resistance-based approaches support functional capacity and inflammatory regulation. Collectively, these findings suggest that individualized program design, structured delivery, and phenotype-informed intervention selection represent practical strategies to address common barriers and enhance implementation feasibility. From a translational perspective, cost-effectiveness considerations remain largely unexplored in the context of combined vitamin D supplementation and exercise interventions. While vitamin D supplementation is generally inexpensive and widely accessible, and structured exercise programs are consistently regarded as cost-effective for the prevention and management of obesity-related cardiometabolic disease, no studies to date have formally evaluated whether combined implementation yields incremental economic benefit beyond exercise alone. Given that current clinical evidence does not consistently demonstrate additive metabolic superiority of vitamin D supplementation in non-deficient populations, the economic justification for routine combined strategies remains uncertain. Future trials should incorporate health economic endpoints to determine whether deficiency-targeted supplementation meaningfully may enhance clinical outcomes in a cost-effective manner.

An additional consideration within the emerging framework of the neurovascular–muscle axis is interindividual variability in responsiveness to vitamin D supplementation. Genetic polymorphisms in key components of vitamin D metabolism and signaling, including VDR, GC (vitamin D binding protein), CYP2R1, and CYP27B1, have been associated with differences in circulating 25(OH)D concentrations and downstream biological effects. These variations may partially explain heterogeneous metabolic and inflammatory responses observed across supplementation trials. However, current evidence remains largely associative and lacks prospective, genotype-stratified interventional validation, particularly in studies integrating structured exercise protocols. As such, genotype-guided personalization of vitamin D dosing cannot yet be recommended in clinical practice. Future randomized trials incorporating genetic profiling and standardized exercise regimens will be essential to determine whether precision-based stratification meaningfully may enhance therapeutic outcomes in individuals with obesity. Although current evidence does not support genotype-guided personalization of vitamin D supplementation or exercise prescriptions, accumulating research suggests that common polymorphisms in the VDR gene may contribute to inter-individual variability in physiological responses. Several studies have identified associations between specific VDR variants (e.g., *Fok*I, *Taq*I, *Bsm*I, and *Apa*I) and phenotypes related to muscle function, body composition, and metabolic traits, implying functional consequences of genetic variation on vitamin D action in target tissues. For example, *Fok*I and *Taq*I polymorphisms have been linked to differential responses in serum 25-hydroxyvitamin D concentrations following supplementation, suggesting a genetic influence on VDR-mediated vitamin D signaling pathways that could modulate effectiveness of nutrient interventions (149). Epidemiological studies report associations between VDR genotypes and measures of muscle mass, strength, and sarcopenia, indicating that genetic variants may shape muscle phenotypes that are relevant to exercise capacity and adaptation (188). Furthermore, observational data have noted links between VDR *Bsm*I, *Taq*I, and *Apa*I genotypes and obesity-related traits, which may reflect genotype-dependent effects on adipose tissue biology and metabolic regulation (189). While these findings remain inconsistent and not yet clinically actionable, they underscore that VDR polymorphisms represent biologically plausible modifiers of individual responses to vitamin D status and potentially to exercise-related outcomes. Consequently, future research integrating genomic analyses with controlled interventions could help clarify how genetic variation in the VDR may inform personalized approaches to optimizing the combined effects of vitamin D and exercise in diverse populations.

This review has several limitations that should be acknowledged. First, the present work was conducted as a narrative review and did not include a formal meta-analysis. Consequently, no meta-regression, moderator analysis, or assessment of publication bias was performed. The included studies were highly heterogeneous in terms of study design (preclinical vs. clinical), participant characteristics, vitamin D dosage, exercise modality, intervention duration, and outcome reporting, which limited the feasibility of pooled quantitative synthesis. Moreover, several studies did not report standardized effect sizes or sufficient statistical parameters necessary for advanced quantitative analyses. Therefore, while this review provides a comprehensive qualitative synthesis of the current evidence, future systematic reviews with harmonized methodologies and consistent outcome reporting are needed to enable robust meta-analytic and moderator-based investigations. Another important limitation of this review is the absence of a systematic analysis of dose–response relationships between vitamin D supplementation and exercise-related outcomes. The included studies used highly variable dosing regimens (e.g., daily, weekly, loading protocols), intervention durations, baseline vitamin D status criteria, and outcome measures. Furthermore, most trials did not directly compare multiple vitamin D doses within the same study design, limiting the ability to evaluate graded effects across dosage levels. The heterogeneity in reporting and study methodology precluded a structured cross-study dose–response synthesis. Therefore, conclusions regarding optimal dosing strategies remain tentative, and future well-controlled trials specifically designed to investigate dose–response relationships are warranted. In conclusion, the combined application of vitamin D supplementation and physical exercise represents a promising, biologically plausible strategy for addressing obesity-related metabolic, musculoskeletal, and neurocognitive dysfunction; however, current human evidence supports predominantly complementary or additive benefits rather than unequivocal synergistic effects. Exercise remains the dominant determinant of improvements in adiposity, insulin sensitivity, and functional capacity, while vitamin D appears to optimize the biochemical and cellular environment that supports these adaptations, particularly in individuals with deficiency. Future research should prioritize well-powered randomized controlled trials specifically designed to test interaction effects between vitamin D and exercise, incorporate standardized dosing strategies, and include mechanistic endpoints spanning muscle, vascular, and neurocognitive domains. Such studies will be essential to determine whether true clinical synergy exists and to translate the neurovascular–muscle axis from a mechanistic construct into an evidence-based therapeutic framework.
